# A20-Deficient Mast Cells Exacerbate Inflammatory Responses *In Vivo*

**DOI:** 10.1371/journal.pbio.1001762

**Published:** 2014-01-14

**Authors:** Klaus Heger, Kaat Fierens, J. Christoph Vahl, Attila Aszodi, Katrin Peschke, Dominik Schenten, Hamida Hammad, Rudi Beyaert, Dieter Saur, Geert van Loo, Axel Roers, Bart N. Lambrecht, Mirjam Kool, Marc Schmidt-Supprian

**Affiliations:** 1 Molecular Immunology and Signal Transduction, Max Planck Institute of Biochemistry, Martinsried, Germany; 2 Laboratory of Immunoregulation, Department of Pulmonary Medicine, University Hospital Ghent, Ghent, Belgium; 3 Department for Molecular Biomedical Research, Vlaams Instituut voor Biotechnologie, Ghent, Belgium; 4 Department of Surgery, Ludwig Maximilians Universität, Munich, Germany; 5 Institute for Immunology, Technische Universität Dresden, Dresden, Germany; 6 Department of Immunobiology, Yale University School of Medicine, New Haven, Connecticut, United States of America; 7 Department of Biomedical Molecular Biology, Ghent University, Ghent, Belgium; 8 II. Medizinische Klinik, Klinikum rechts der Isar, Technische Universität München, Munich, Germany; 9 Department of Pulmonary Medicine, Erasmus University Medical Center, Rotterdam, The Netherlands; NIAMS/NIH, United States of America

## Abstract

Mast cells are implicated in the pathogenesis of inflammatory and autoimmune diseases. However, this notion based on studies in mast cell-deficient mice is controversial. We therefore established an *in vivo* model for hyperactive mast cells by specifically ablating the NF-κB negative feedback regulator A20. While A20 deficiency did not affect mast cell degranulation, it resulted in amplified pro-inflammatory responses downstream of IgE/FcεRI, TLRs, IL-1R, and IL-33R. As a consequence house dust mite- and IL-33-driven lung inflammation, late phase cutaneous anaphylaxis, and collagen-induced arthritis were aggravated, in contrast to experimental autoimmune encephalomyelitis and immediate anaphylaxis. Our results provide *in vivo* evidence that hyperactive mast cells can exacerbate inflammatory disorders and define diseases that might benefit from therapeutic intervention with mast cell function.

## Abstract 2

Mast cells, best known as effector cells in pathogenic immunoglobulin-mediated responses, can sense a variety of “danger” signals; if manipulated to enhance their resulting inflammatory responses, they also exacerbate inflammatory diseases such as arthritis and lung inflammation.

## Author Summary

Mast cells mediate allergic and anaphylactic immune reactions. They are also equipped with innate pattern recognition, cytokine, and alarmin receptors, which induce inflammatory responses. Correlative studies in human patients hinted at roles for mast cells in autoimmune and inflammatory diseases. However, studies using mast cell-deficient mice have yielded contradictory results in this context. In this study we determined that A20, the negative feedback regulator, restricts inflammation downstream of the mast cell antigen (allergen) receptor module, innate pattern recognition receptors, and the alarmin receptor IL-33R. By mast cell–specific ablation of A20 we established a mouse model for exaggerated inflammatory but normal anaphylactic mast cell signaling. With these mice we evaluated the impact of increased mast cell-mediated inflammation under experimental conditions aimed at mimicking several inflammatory human diseases. Our results demonstrated that the lack of A20 from mast cells exacerbated disease in mouse models for rheumatoid arthritis and innate forms of asthma, but did not impact disease progression in a mouse model for multiple sclerosis. Our data provide direct evidence that enhanced inflammatory mast cell responses can contribute to disease pathology and do so via sensing and amplifying local inflammatory reactions driven by “danger” stimuli and/or tissue damage that leads to the release of alarmins.

## Introduction

Mast cells are innate immune cells that localize preferentially to vascularized tissues at the host-environment barrier. Through their high-affinity IgE receptor (FcεRI) they can capture circulating IgE and are hence primed to degranulate and produce cytokines upon antigen encounter. Mast cells store large amounts of histamine, heparin, and various proteases, which they release within minutes during degranulation. In contrast, the release of pro-inflammatory lipid mediators and most cytokines requires *de novo* synthesis upon activation [1],[2]. Mast cells are also equipped with a range of cell surface receptors allowing them to sense microbial invasion, inflammation, and tissue damage, among them several TLRs, the IL-1R, and the receptor for the alarmin IL-33, the IL-33R. Engagement of these receptors initiates a pro-inflammatory gene expression program via NF-κB transcription factors [1],[3],[4].

More than 100 years after their discovery, the physiological roles of mast cells in health and disease remain heavily disputed [5]. It is widely accepted that they are central mediators of IgE-dependent allergic responses, which can cause life-threatening anaphylactic shock in susceptible individuals [2]. These deleterious mast cell properties could be overshooting, misdirected responses originally designed as components of allergic host defense against environmental irritants, noxious foreign substances, and envenomation [6]. Although these functions might explain the evolutionary pressure that led to the development and preservation of mast cells, it is generally believed that further protective properties await identification. Furthermore, the observation that human patients suffering from asthma, allergic rhinitis, atopic dermatitis, and autoimmune and malignant disorders consistently contained mast cell accumulations at affected locations indicated a role for mast cells in these diseases [3],[7].

Mouse strains lacking mast cells due to different loss-of-function mutations in the receptor tyrosine kinase c-Kit were instrumental to elucidate mast cell *in vivo* functions. However, in the context of autoimmune, inflammatory, allergic, and malignant disease studies, these mouse strains often yielded conflicting results, presumably due to additional effects of c-Kit deficiency [5]. Moreover, recent experiments employing novel Kit-independent mast cell-deficient mouse models have challenged some of their initially proposed functions [8]–[10]. Regardless of the particular model, loss-of-function approaches describe the consequences of absent function, which is not always inversely correlated with excessive function, and functional compensation by other cell types can be a problem. We aimed to establish a new mouse strain modeling gain-of-function of inflammatory mast cell responses, as they are at the center of controversy.

In various immune lineages, the ubiquitin-editing enzyme and NF-κB negative feedback regulator A20 (also known as Tnfaip3) is critical for the prevention of inflammation and autoimmunity [11]–[16]. Polymorphisms in the *A20* gene locus or its binding partner *TNIP1* are significantly associated with a number of human inflammatory and autoimmune conditions [17],[18] and in case of *TNIP1* also with asthma [19]. Therefore, we postulated that A20 deficiency in mast cells should provide an ideal genetic model system to address their pro-inflammatory properties in a gain-of-function approach. We employed conditional gene ablation to demonstrate that A20 restricts NF-κB activation downstream of the IgE:FcεRI module, TLRs, the IL-1R, and the IL-33R in mast cells. Exaggerated signaling from these receptors to NF-κB strongly enhanced mast cell pro-inflammatory responses, but did not affect degranulation. The presence of these hyperactive inflammatory mast cells exacerbated late phase cutaneous anaphylaxis reactions, allergic lung, and autoimmune joint inflammation. Our findings are, to our knowledge, the first direct demonstration that enhanced inflammatory mast cell responses can contribute to disease pathology.

## Results

### A20 Is a Negative Regulator of TLR-, IL-33R-, and IgE/FcεRI-Induced NF-κB Activation in Mast Cells

Upon priming with IgE and subsequent aggregation, the mast cell FcεRI activates NF-κB in a similar way to B and T cell antigen receptors [20], whereas the receptor for IL-33 initiates signaling cascades analogous to TLR and IL-1R engagement via MyD88 and TRAF6 [4]. We thus hypothesized that in analogy with other immune cells [18], induced expression of A20 could restrict NF-κB activation downstream of the IgE/FcεRI module and the IL-33R, as well as the IL-1R and TLRs in mast cells.

**Figure 1:**
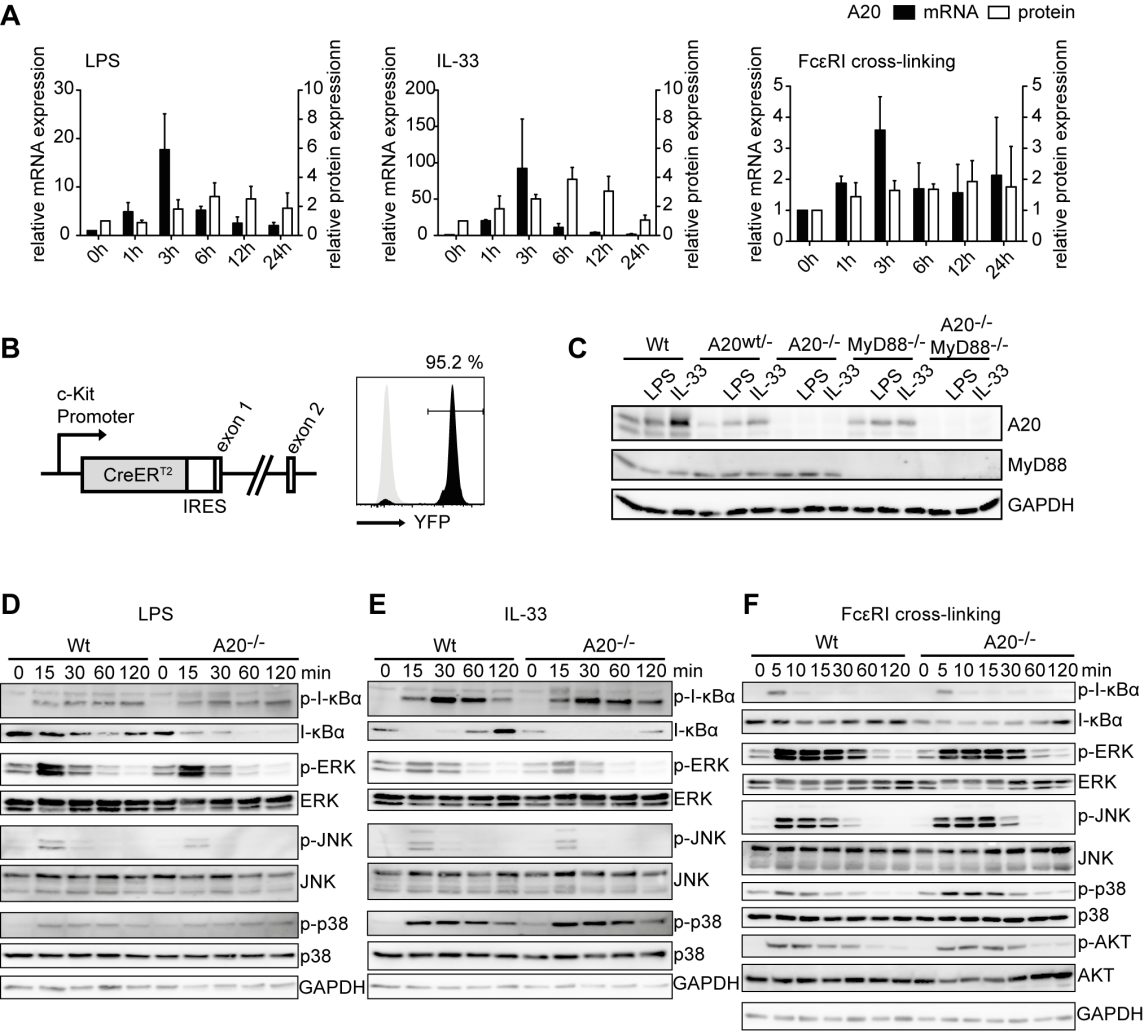
A20 is a specific negative feedback regulator of NF-κB activation in mast cells. (A) Wild-type BMMCs were stimulated with 10 µg/mL LPS or 10 ng/mL IL-33 for the indicated time intervals. To induce FcεRI cross-linking, BMMCs were loaded overnight with 1 µg/mL anti-DNP IgE and subsequently stimulated with 10 ng/mL DNP–HSA. A20 mRNA and protein levels were determined by quantitative RT-PCR and Western blotting, respectively. Changes in mRNA levels normalized to PBGD (black bars) and protein levels normalized to GAPDH (white bars) relative to time-point 0 h are shown. Data are means + SD from three independent experiments. (B) Schematic representation of the *Kit^CreERT2^* knock-in allele. Black and grey histograms show representative YFP expression in BMMCs derived from *Kit^CreERT2/+^R26-Stop^F^YFP* and control animals, respectively, treated for 7 d with 1 µM 4-hydroxytamoxifen. (C) Western blot of whole BMMC lysates of the indicated genotypes unstimulated or activated for 3 h with 10 µg/mL LPS or 10 ng/mL IL-33. Data are representative of three independent experiments. (D–F) Western blots of whole BMMC lysates stimulated for the indicated time intervals with 10 µg/mL LPS (D), 10 ng/mL IL-33 (E), or loaded for 2 h with 1 µg/mL anti-DNP IgE and subsequently stimulated with 10 ng/mL DNP–HSA (F). Data are representative of at least two independent experiments.

To biochemically address this hypothesis, we first investigated A20 expression kinetics in murine bone-marrow-derived mast cells (BMMCs) upon activation by LPS, IL-1β, IL-33, and FcεRI cross-linking. A20 transcript levels peaked 3 h after activation and declined steadily afterwards, with IL-33 being the most potent inducer (Figure 1A and Figure S1A). A20 protein levels increased 3–6 h after activation and remained elevated for up to 12 h (Figure 1A and Figure S1B). In order to avoid potential biases through effects of gene deficiencies during mast cell *in vitro* development, we employed the novel *Kit^CreERT2^* transgene [21],[22]. This allowed very efficient inducible expression of a fluorescent reporter protein (Figure 1B), and excision of conditional *A20* and *MyD88* alleles in BMMCs (Figure 1C) did not affect c-Kit and FcεRI levels (Figure S1C). A20-deficient mast cells activated by LPS, IL-33, and FcεRI cross-linking showed enhanced activation of NF-κB as indicated by prolonged degradation and delayed resynthesis of its inhibitor I-κBα, while no differences in the activation of MAPK signaling were observed (Figure 1D–F and Figure S1D).

Collectively, these results show that A20 is a central negative feedback regulator of NF-κB signaling and mast cell activation in response to TLR ligands, IL-33, and antigen/IgE complexes.

### Mast Cell-Specific Ablation of A20 *in Vivo* Does Not Induce Spontaneous Pathology

We observed dramatically enhanced NF-κB activation in A20-deficient mast cells in response to various physiologically relevant stimuli *in vitro*. Therefore, we generated mice lacking A20 specifically in connective tissue-type mast cells (*Mcpt5Cre A20^F/F^*) [16],[23]. In order to specifically dissect innate MyD88-dependent from other signals, we generated mice containing mast cells deficient for both A20 and MyD88 (*Mcpt5Cre A20^F/F^MyD88^F/F^*) [24]. Most experiments were controlled with *Mcpt5Cre* transgenic animals and, to a lesser extent, with nontransgenic littermates. As we did not observe any differences between those two groups, they are shown together as control mice.

**Figure 2:**
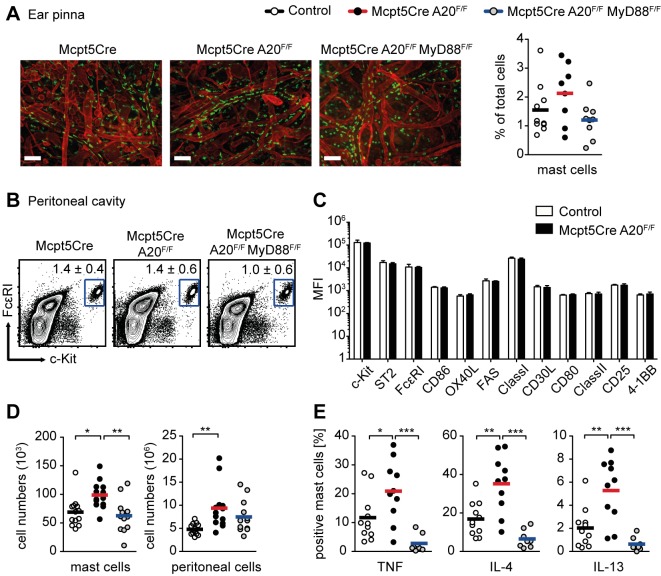
*In vivo* homeostasis of A20-deficient mast cells. (A) Representative immunofluorescence images of whole-mount ear explants: green, avidin-FITC; red, anti-laminin; scale bar, 100 µm. Scatter plot shows proportions of mast cells (CD45^+^FcεRI^+^c-Kit^+^) in ear skin digests as identified by flow cytometric analysis. Bars indicate means from at least eight mice per genotype (Control, 7 *Mcpt5Cre*, 3 Cre^−^ littermates). (B) Dot plots showing proportions of ex vivo isolated peritoneal mast cells (FcεRI^+^c-Kit^hi^). Numbers represent means ± SD from at least 20 mice per genotype (Control, 15 *Mcpt5Cre*, 5 Cre^−^ littermates). (C) Median fluorescent intensities (MFIs) of various cell surface markers expressed on A20-deficient and control *ex vivo* isolated peritoneal mast cells. Data are means + SD from at least four mice per genotype (Control, 4 *Mcpt5Cre*, 1 Cre^−^ littermate). (D) Scatter plots show absolute numbers of peritoneal mast cells (FcεRI^+^c-Kit^hi^) and total peritoneal cells. Bars indicate means from at least 10 mice per genotype (Control, 8 *Mcpt5Cre*, 5 Cre^−^ littermates). (E) Scatter plots show proportions of cytokine positive peritoneal mast cells (c-Kit^+^) as identified by intracellular flow cytometric analysis. Bars indicate means from at least eight mice per genotype (Control, 9 *Mcpt5Cre*, 2 Cre^−^ littermates). *p<0.05, **p<0.01, ***p<0.001 (one-way ANOVA).

*Mcpt5Cre A20^F/F^* mice developed normally, showing no macroscopic signs of disease (unpublished data). A20-deficient mast cells homed to their natural positions in the dermis and normal proportions were located in close proximity to blood vessels (Figure 2A). Mast cell numbers in the ear and dorsal skin were not significantly altered in *Mcpt5Cre A20^F/F^* compared to control mice (Figure 2A and Figure S2A). A20 deficiency did not affect the proportions (Figure 2B) or surface phenotype (Figure 2C) of mast cells in the peritoneal cavity. However, absolute mast cell numbers were significantly increased in *Mcpt5Cre A20^F/F^* mice in comparison to controls, due to an overall increase in peritoneal cellularity (Figure 2D). Furthermore, we observed enhanced frequencies of TNF-, IL-4–, and IL-13–producing peritoneal mast cells in *Mcpt5Cre A20^F/F^* in comparison to control mice (Figure 2E and Figure S2B). Ablation of MyD88 in addition to A20 led to a normalization of mast cell numbers (Figure 2D) and cytokine production (Figure 2E and Figure S2B). Complete ablation of A20 and MyD88 was confirmed by Western blotting of peritoneal cavity-derived mast cell (PMC) cultures (Figure S2C). To confirm that the *Mcpt5Cre* transgene does not lead to A20 deletion in cell types other than mast cells, we performed quantitative probe-based real-time PCR on genomic DNA from cell populations purified from *Mcpt5Cre A20^F/F^* animals. Using this sensitive approach we determined that less than 0.1% of A20-deficient cells are contained among sorted leukocytes (T and B cells) and various myeloid cell populations (Figure S2D), if any. Interestingly, analysis of secondary lymphoid organs revealed minor splenomegaly in *Mcpt5Cre A20^F/F^* mice in comparison to controls, which depended on signals via MyD88 (Figure S2E).

Our results thus indicate that MyD88-dependent signals *in vivo* induce a preactivated or poised state in A20-deficient mast cells without causing spontaneous general inflammation.

### A20 Deficiency Prolongs Survival and Enhances Proliferation of Activated Mast Cells

*Mcpt5Cre A20^F/F^* mice showed elevated mast cell numbers in the peritoneal cavity. This could be due to enhanced mast cell survival as A20 has also been implicated in cell death responses [13],[15],[16],[18]. To clarify the function of A20 in mast cell survival, we studied growth factor deprivation-induced apoptosis, which in BMMCs is antagonized by prosurvival members of the Bcl-2 family including the NF-κB target genes Bcl-x_L_ and A1 [25]. As A20-deficient mast cells showed stronger NF-κB activation in response to stimulation, we reasoned that loss of A20 could protect activated mast cells from IL-3 and SCF withdrawal-induced cell death.

**Figure 3:**
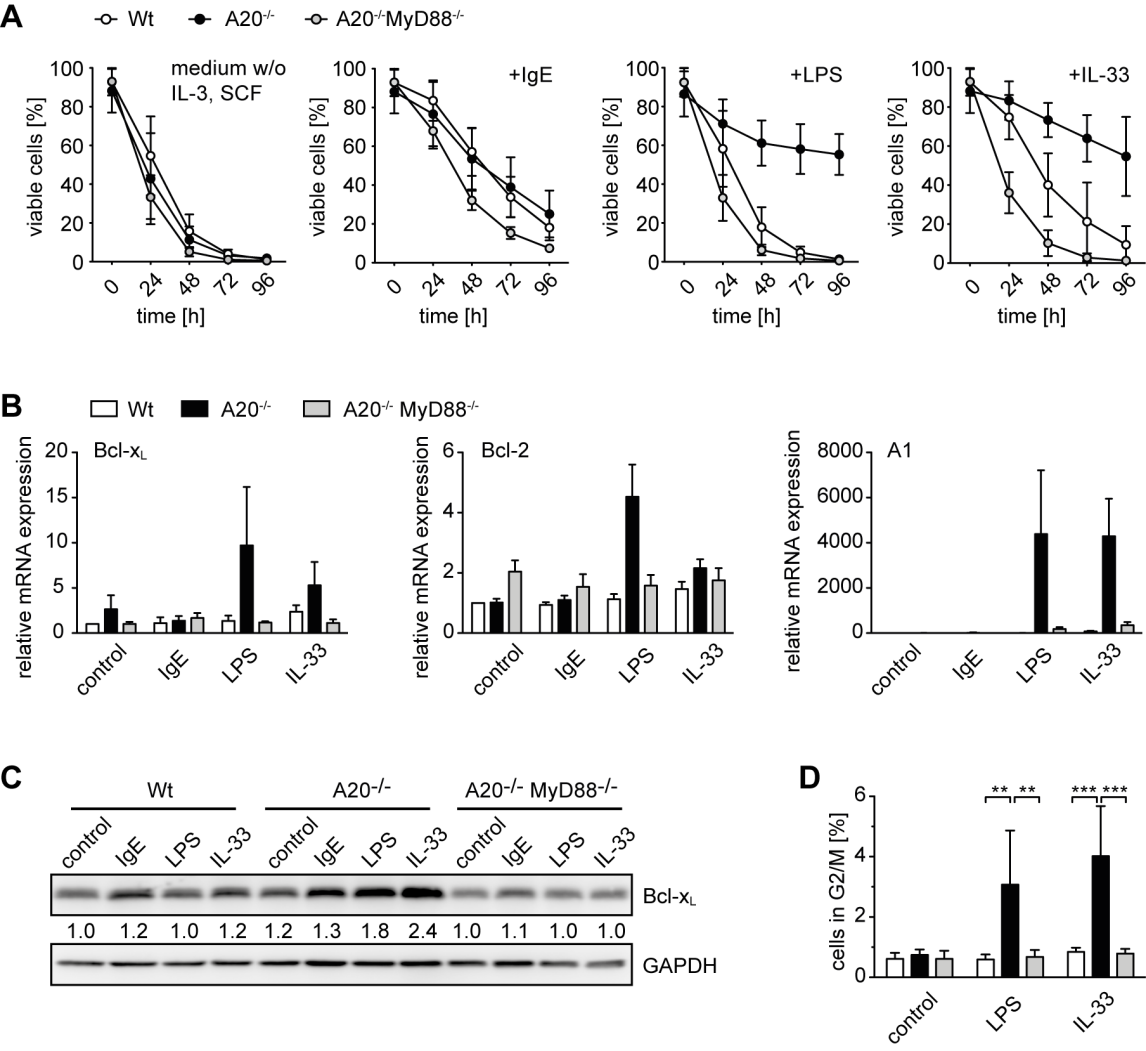
A20 deficiency enhances survival and proliferation of LPS- and IL-33-activated mast cells. (A) Survival kinetics of IL-3 and SCF-deprived BMMCs cultured in simple medium or supplemented with 5 µg/mL anti-DNP IgE, 10 µg/mL LPS, or 10 ng/mL IL-33. Cell death was quantified at the indicated time points after propidium iodide staining and flow cytometric analysis. Data are means ± SD from at least three independent experiments. (B) RNA was purified from BMMCs treated for 24 h as described in (A) and Bcl-2, Bcl-x_L_, and A1 mRNA levels were determined by quantitative RT-PCR. Relative changes in transcript levels to unstimulated (Bcl-x_L_ and Bcl-2) or IgE-stimulated (A1) wild-type BMMCs were calculated after normalization for PBGD. Data are means + SD from three independent experiments. (C) Western blot of whole BMMC lysates treated for 24 h as described in (A). Numbers denote changes in Bcl-x_L_ protein levels relative to unstimulated wild-type BMMCs. Data are representative of and numbers are means from three independent experiments. (D) Cell cycle profile analysis by propidium iodide staining of BMMCs cultured for 4 d in full medium alone or supplemented with 10 µg/mL LPS or 10 ng/mL IL-33. Percentages of live cells in G2/M phase are shown and are means + SD from two independent experiments with two independent mast cell preparations. **p<0.01, ***p<0.001 (one-way ANOVA).

A20 deficiency *per se* did not affect growth factor deprivation-induced cell death, and stimulation with monomeric IgE generally enhanced mast cell survival by an A20-independent pathway (Figure 3A). However, treatment with LPS remarkably increased survival only of mast cells lacking A20. IL-33 had a small effect on wild-type mast cells but profoundly inhibited cell death in the absence of A20 (Figure 3A). Stimulation with LPS or IL-33 enhanced the transcription of all tested pro-survival members of the Bcl-2 family in A20-deficient cells in comparison to controls, with A1 being most prominently affected (Figure 3B). Enhanced expression of Bcl-x_L_ protein was confirmed by Western blotting (Figure 3C). All these processes were strictly MyD88-dependent (Figure 3A–C). We thus conclude that A20 deficiency increases the up-regulation of pro-survival Bcl-2 family members upon innate activation. This is one potential mechanism of how loss of A20 can lead to a dramatically enhanced protection against apoptosis. In addition, cell cycle analysis showed an increase in proliferative activity of LPS and IL-33–stimulated A20-deficient mast cells, which depended on signals transmitted via MyD88 (Figure 3D).

Collectively, these results indicate that naturally occurring MyD88-transmitted stimuli can promote the survival and proliferation of A20-deficient mast cells.

### Loss of A20 in Mast Cells Enhances Allergic Airway Inflammation

**Figure 4:**
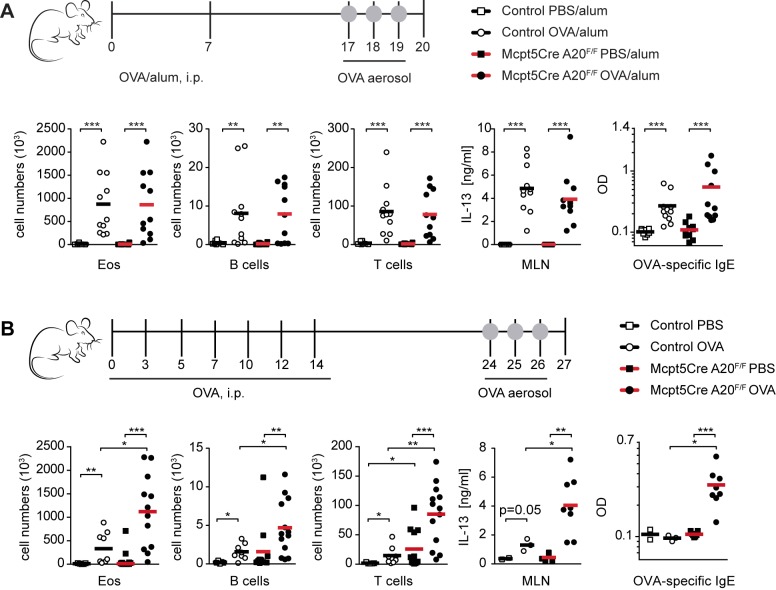
Enhanced OVA-induced airway inflammation in *Mcpt5Cre A20^F/F^* mice in the absence of alum. (A) Scatter plots show absolute BAL fluid cell numbers of Eos (eosinophils, SSC^hi^, Siglec-F^+^, Gr-1^int^, CD11c^−^), B cells (FSC/SSC^lo^, CD19^+^, MHCII^+^), and T cells (FSC/SSC^lo^, CD3^+^, MHCII^−^), as identified by flow cytometric analysis. IL-13 cytokine production by MLN cells and OVA-specific IgE levels were measured by ELISA. Bars indicate means; data are integrated from two experiments and are representative of three independent experiments with four to eight mice per group (Control, 8 “PBS/alum” and 11 “OVA/alum” Cre^−^ littermates). (B) Scatter plots show absolute BAL fluid cell numbers of Eos (eosinophils, SSC^hi^, Siglec-F^+^, Gr-1^int^, CD11c^−^), B cells (FSC/SSC^lo^, CD19^+^, MHCII^+^), and T cells (FSC/SSC^lo^, CD3^+^, MHCII^−^), as identified by flow cytometric analysis. IL-13 cytokine production by MLN cells and OVA-specific IgE levels were measured by ELISA. Bars indicate means, and data are integrated from two independent experiments with two to eight mice per group (Control, 5 “PBS” and 8 “OVA” Cre^−^ littermates). *p<0.05, **p<0.01, ***p<0.001 (Mann-Whitney test).

Next we investigated the consequences of mast cell-specific A20 deficiency in allergic, inflammatory, and autoimmune conditions. Asthma is a chronic inflammatory disease of the airways characterized by increased presence of eosinophils and production of Th2 cytokines, such as IL-13, that causes bronchial hyperreactivity and goblet cell metaplasia. The classical mouse asthma model, which is induced by injection of ovalbumin (OVA) together with alum adjuvant, is mast cell independent in *Kit^W/Wv^* and *Kit^W-sh/W-sh^* mice. In contrast, a clear role for mast cells has been demonstrated in models of asthma that employ OVA as an allergen in the absence of alum [26],[27]. To address the effect of mast cell-specific A20 deficiency in mouse asthma models, we immunized mice with OVA either in the presence or absence of alum, and challenged mice 10 d later with 1% OVA aerosols. In the OVA/alum model, loss of A20 in mast cells did not affect the number of bronchoalveolar lavage (BAL) fluid eosinophils, B and T cells, and the production of IL-13 by mediastinal lymph node (MLN) mononuclear cells or serum levels of OVA-specific IgE (Figure 4A). In contrast, when mice were actively sensitized to OVA in the absence of alum, all these parameters were significantly elevated in *Mcpt5Cre A20^F/F^* compared to control mice (Figure 4B).

**Figure 5:**
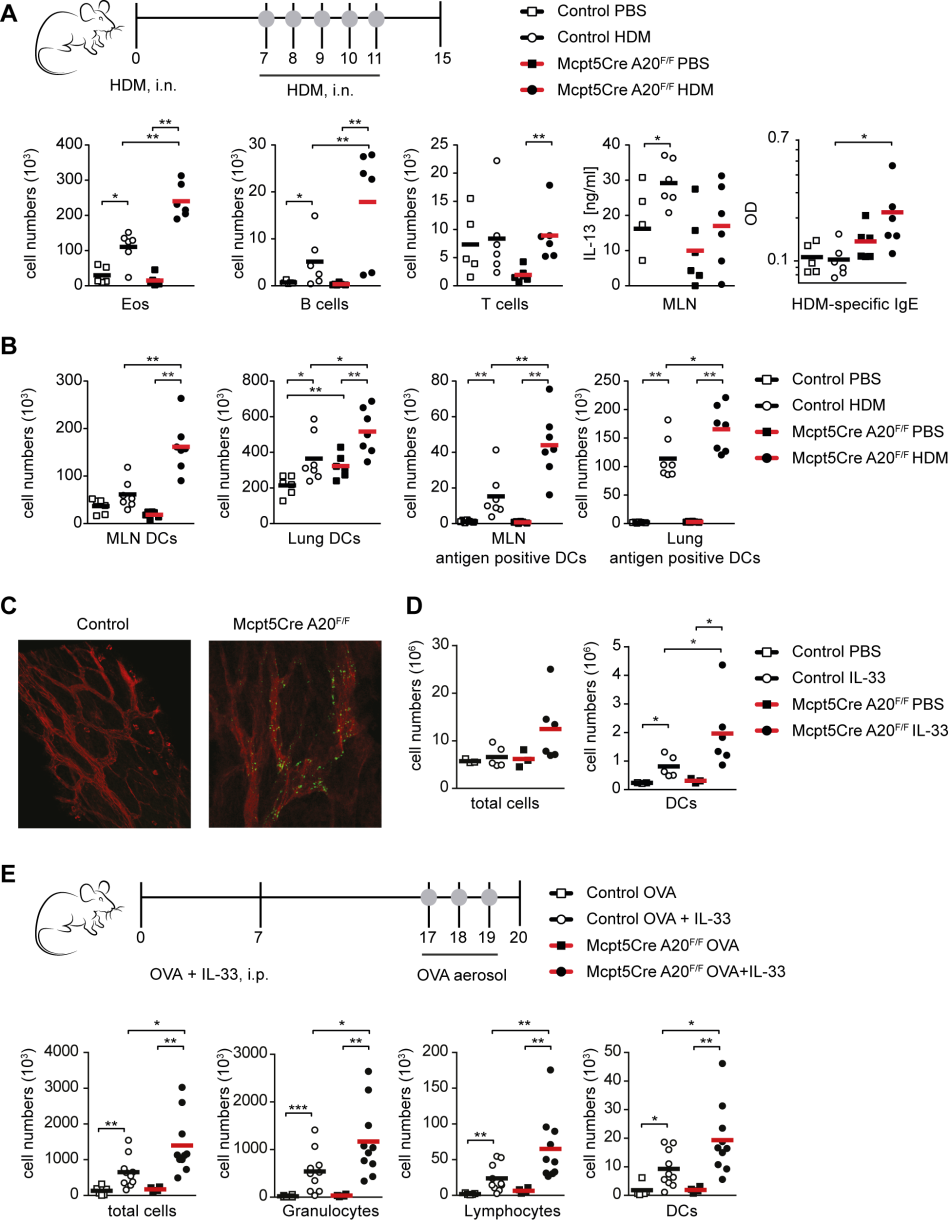
Enhanced inflammation in *Mcpt5Cre A20^F/F^* mice in the HDM, IL-33, and OVA + IL-33 asthma models. (A) Scatter plots show absolute BAL fluid cell numbers of Eos (eosinophils, SSC^hi^, Siglec-F^+^, Gr-1^int^, CD11c^−^), B cells (FSC/SSC^lo^, CD19^+^, MHCII^+^), and T cells (FSC/SSC^lo^, CD3^+^, MHCII^−^), as identified by flow cytometric analysis. Cytokine production by MLN cells and HDM-specific IgE levels were measured by ELISA. Bars indicate means, and data are representative of three independent experiments with four to six mice per group (Control, 5 “PBS” and 6 “HDM” Cre^−^ littermates). (B) Mice received 100 µg HDM extract and 10 µg OVA-AF647 intratracheally and were analyzed 18 h later. Scatter plots show absolute numbers of DCs (DCs, CD11c^+^, MHCII^hi^, nonautofluorescent) and antigen positive DCs (AF647^+^) in the lung and MLNs as identified by flow cytometric analysis. Bars indicate means from six to seven mice per group (Control, 6 “PBS” and 7 “HDM” Cre^−^ littermates). (C) Representative confocal images of trachea whole mounts of HDM sensitized and challenged mice after i.v. injection of FITC^+^ 500 nm microbeads: green, FITC^+^ microbeads; red, anti-CD31. (D) Mice were administered 100 ng IL-33 i.n. on 3 consecutive days. Scatter plots show absolute lung cell numbers and DCs (CD11c^+^, MHCII^hi^, nonautofluorescent) as identified by flow cytometric analysis. Bars indicate means from three to six mice per group (Control, 3 “PBS” and 5 “IL-33” Cre^−^ littermates). (E) Scatter plots show absolute BAL fluid cell numbers, granulocytes (SSC^int→hi^, Ly6C^+^, Ly6G^int→hi^, CD11b^+^, CD11c^−^), lymphocytes (FSC/SSC^lo^, CD3^+^, or CD19^+^), and DCs (CD11c^+^, MHCII^hi^, nonautofluorescent) as identified by flow cytometric analysis. Bars indicate means, and data are integrated from two independent experiments with two to six mice per group (Control, 5 “OVA” and 10 “OVA + IL-33” Cre^−^ littermates). *p<0.05, **p<0.01 (Mann-Whitney test).

House dust mite (HDM) allergens are the most common triggers of allergic asthma and robustly induce IgE-dependent lung inflammation with many features of human asthma in mice [28]–[30]. To our knowledge, the role of mast cells has not yet been genetically addressed in this arguably more relevant model. Thus we measured the responses of *Mcpt5Cre A20^F/F^* and control mice to active sensitization induced by administration of HDM extracts via the nasal route followed by five HDM challenges 7 d later. Mast cell-specific ablation of A20 caused increased levels of BAL fluid eosinophils and B cells, and HDM-specific serum IgE (Figure 5A). Dendritic cells (DCs) induce and maintain Th2 immunity to inhaled allergens such as HDM and OVA [31] and are necessary and sufficient for the development of asthma [29]. Suto et al. showed that mast cells control the activation of DCs via the release of TNF [32]. We therefore evaluated whether A20 deficiency in mast cells affects DC responses to HDM exposure. Indeed HDM treatment led to enhanced recruitment of DCs to the lung and MLNs in *Mcpt5Cre A20^F/F^* mice in comparison to controls. In addition, these DCs had taken up significantly more fluorescent antigen (Figure 5B). These findings are in line with our observations that A20-deficient mast cells produce more TNF upon activation. As mast cells have been shown to control vascular permeability [9], we wondered whether the increase in airway inflammation in *Mcpt5Cre A20^F/F^* mice could also be linked to enhanced vascular leakage. Therefore, we injected fluorescently labeled 500 nm microspheres intravenously 1 h after HDM allergen challenge and measured their extravasation 5 min later. In line with our hypothesis, *Mcpt5Cre A20^F/F^* mice displayed a strong increase in vascular leakage compared to control mice (Figure 5C). We and others have previously reported that the HDM-driven model of asthma depends on the activity of IL-33 and is driven by antigen-presenting DCs [28],[33]. In control mice, three intranasal administrations of IL-33 induced an increase in the total cell and DC influx into the lungs, which was enhanced by ablation of A20 specifically in mast cells (Figure 5D). In addition, there was a trend towards increased numbers of eosinophils, neutrophils, and monocytes in the lungs of *Mcpt5Cre A20^F/F^* mice (Figure S3). Hence, the increased sensitivity of A20-deficient mast cells towards IL-33 could contribute to the enhanced allergic responses observed in *Mcpt5Cre A20^F/F^* mice. This notion is supported by the fact that *Mcpt5Cre A20^F/F^* mice showed significantly elevated numbers of granulocytes, lymphocytes, and DCs in the BAL fluid after OVA aerosol challenge, when the OVA sensitization occurred in the presence of IL-33 (Figure 5E). We employed the above-described PCR assay (see Figure S2D) to confirm that under inflammatory conditions, *Mcpt5Cre*-mediated recombination of loxP-flanked A20 alleles is still restricted to mast cells. We did not detect recombination of conditional alleles in the following cellular subsets purified and pooled from eight HDM-challenged *Mcpt5Cre A20^F/F^* mice, which exceeded the background signal detected in the corresponding population purified and pooled from eight *A20^F/F^* mice: Lung B cells, DCs, monocytes and neutrophils, BAL fluid eosinophils, and peritoneal cavity macrophages (unpublished data).

In summary, we show that enhanced connective tissue-type mast cell responses to allergens and the alarmin IL-33 significantly aggravate allergic lung inflammation.

### A20-Deficient Mast Cells Aggravate Collagen-Induced Arthritis (CIA) But Not Experimental Autoimmune Encephalomyelitis (EAE)

We showed that A20-deficient mast cells worsen inflammation during allergic airway responses. Mast cells were also implicated in autoimmune diseases such as multiple sclerosis and arthritis [7],[34]. However, the absence of mast cells had contradicting effects during the induction of model diseases in the mouse [5],[8],[35]–[37]. Hence we revisited this important issue in our novel gain-of-function model.

**Figure 6:**
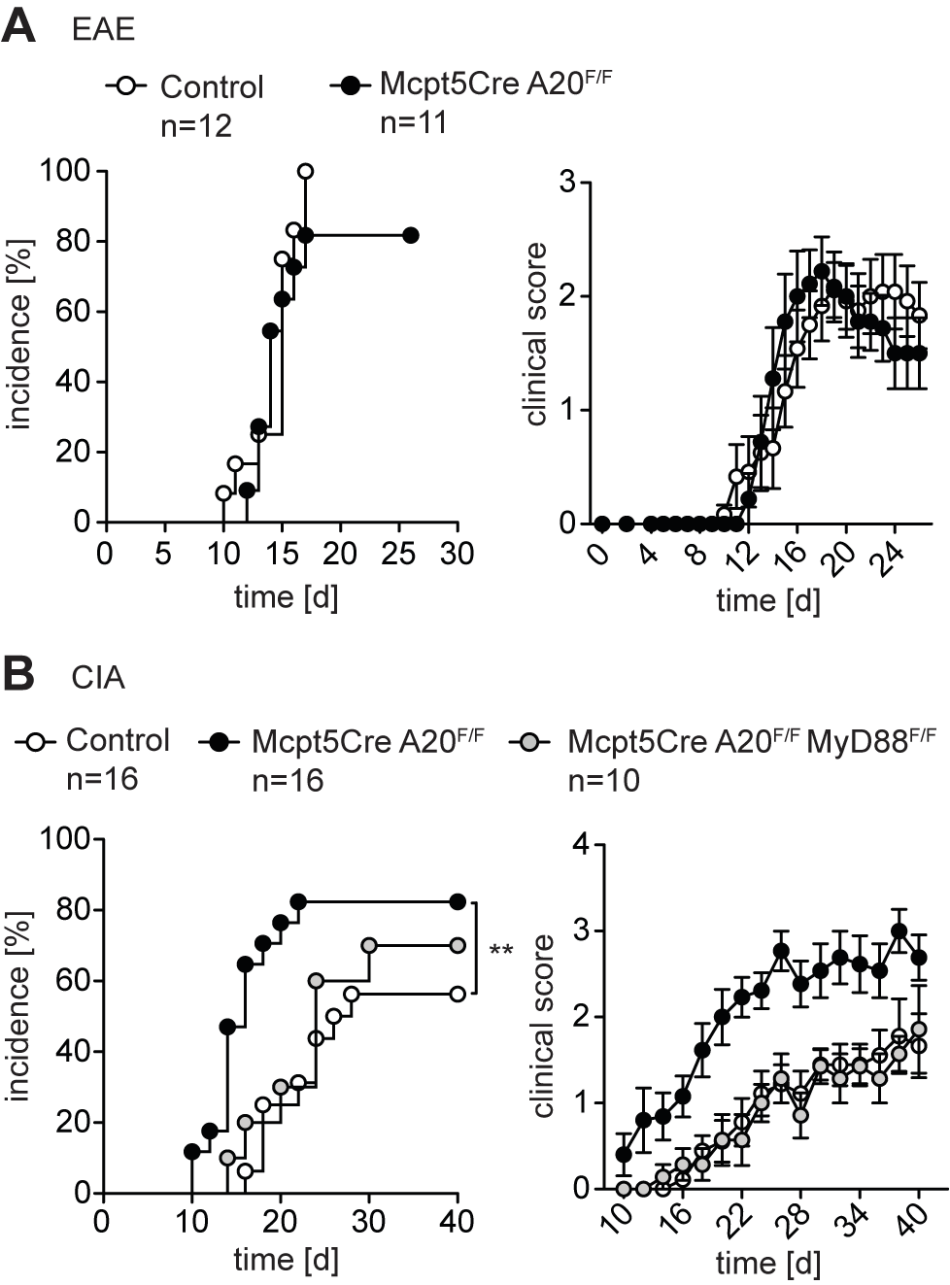
Enhanced joint but normal nervous system inflammation in *Mcpt5Cre A20^F/F^* mice. (A) EAE disease incidence was assessed and severity scored every day based on clinical symptoms. Data are mean scores of sick animals ± SEM (for visual clarity instead of SD) from at least 11 mice per genotype (Control, 12 *Mcpt5Cre* mice). (B) CIA disease incidence was assessed and severity scored every second day based on clinical symptoms. Data are mean scores of sick animals ± SEM (for visual clarity instead of SD) from at least 10 mice per genotype (Control, 16 *Mcpt5Cre* mice). **p<0.01 (Log-rank (Mantel-Cox) test).

We induced EAE through standard protocols in control and *Mcpt5Cre A20^F/F^* mice. Monitoring of disease incidence as well as severity (clinical score) did not yield any differences between both groups (Figure 6A). Thus, A20-deficient connective tissue-type mast cells do not influence T-cell-driven EAE.

Both genetic association studies and experimental approaches in mouse models suggested that mast cells and A20 play a pathological role in rheumatoid arthritis [12],[18],[34]. Interestingly, many studies showed a pathological role of IL-33 in CIA [38],[39]. In line with our previous results, suggesting an important role for A20 in controlling IL-33R signaling, *Mcpt5Cre A20^F/F^* mice exhibited an earlier onset of disease after immunization compared to control mice (Figure 6B). Also disease incidence and severity, as assessed by clinical score and paw swelling (Figure 6B and Figure S4A), were significantly exacerbated by A20 deficiency in mast cells. Evaluation of pathology by histology corresponded well with the clinical scores (Figure S4B). Systemically, we detected slightly elevated levels of TNF (Figure S4C) and an expansion of splenic B and T cell numbers in *Mcpt5Cre A20^F^* ^/F^ mice, pointing to increased inflammation (Figure S4D). Concomitant ablation of MyD88 reversed the effects of mast cell-specific A20 loss during arthritis induction to a large extent (Figure 6B and Figure S4A–D), indicating essential roles for IL-33, TLR ligands, and/or IL-1β.

Taken together, our data show that A20-deficient connective tissue-type mast cells did not affect EAE, but increased the severity of arthritis-associated inflammation.

### A20 Deficiency Does Not Affect Immediate But Exacerbates Late Phase Anaphylactic Mast Cell Responses

**Figure 7:**
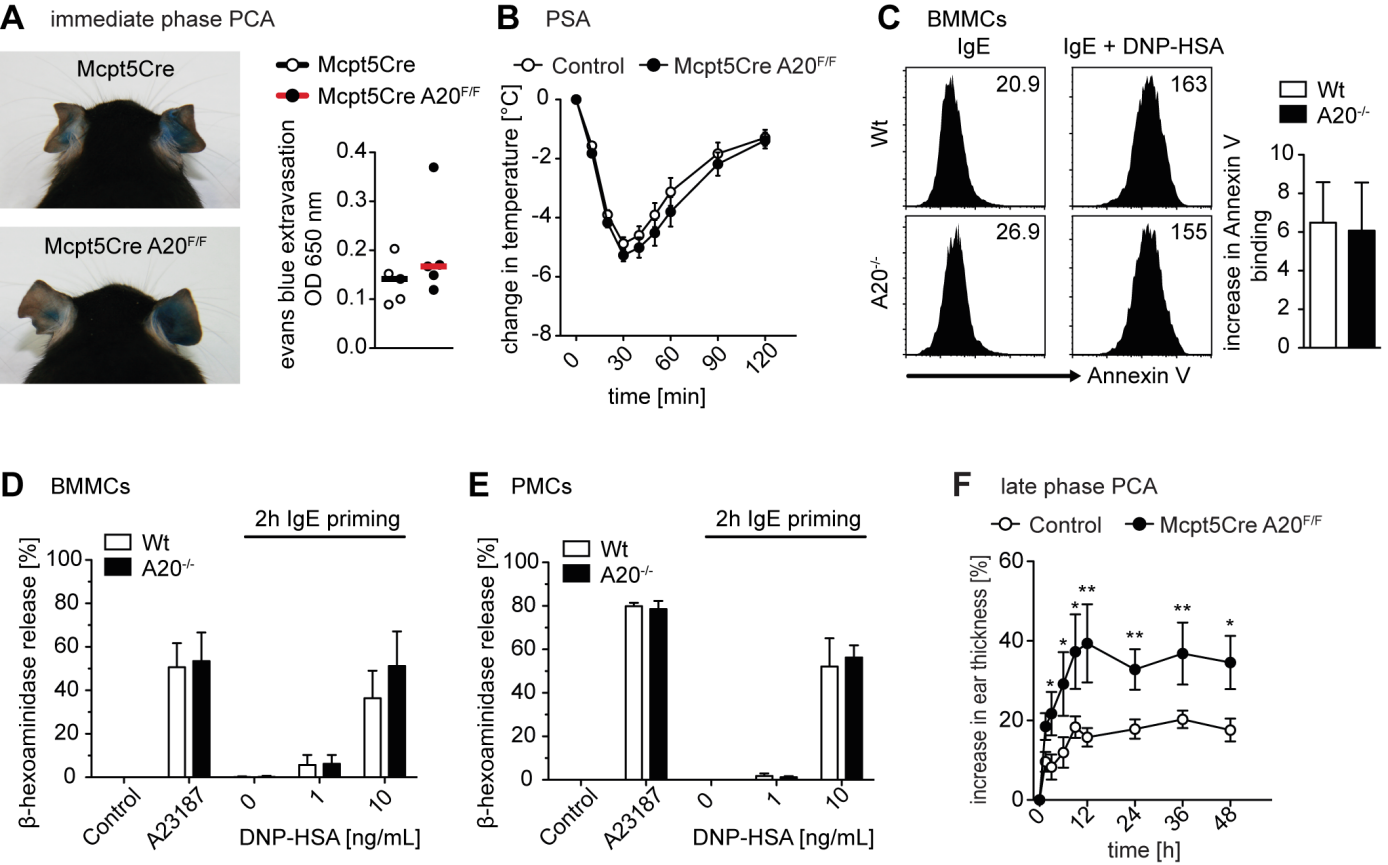
Normal degranulation and immediate but exacerbated late phase anaphylactic responses in the absence of A20. (A) Representative images of immediate phase PCA reactions (PBS, left ear; anti-DNP IgE, right ear). Evans blue extravasation was quantified by extraction and photometric analysis. Data show differences in dye extravasation between IgE and PBS injected ears, and bars are medians from five mice per genotype (Control, 5 *Mcpt5Cre* mice). (B) Data show changes in rectal temperature over time during PSA reactions and are means ± SEM (for visual clarity instead of SD) from at least 22 mice per genotype (Control, 7 *Mcpt5Cre* and 16 Cre^−^ littermates). (C) BMMCs were loaded for 2 h with 1 µg/mL anti-DNP IgE and subsequently stimulated for 20 min with 10 ng/mL DNP-HSA, stained with Annexin V-Cy3, and analyzed by flow cytometry. Representative histograms including MFIs and fold increase in Annexin V binding are shown comparing IgE loaded with degranulated BMMCs. Data are means + SD from at least five independent mast cell preparations. (D and E) BMMCs (D) or PMCs (E) were loaded as in (C) and subsequently stimulated for 30 min with the indicated concentrations of DNP–HSA or 500 ng/mL A23187. Degranulation was determined by measuring the activity of β-hexosaminidase in supernatants and cell lysates. Data are means + SD from seven (D) or three (E) independent mast cell preparations. (F) Data show increase in ear thickness during late phase PCA reactions and are means ± SEM (for visual clarity instead of SD) from at least seven mice per genotype (Control, 8 *Mcpt5Cre* and 13 Cre^−^ littermates). *p<0.05, **p<0.01 (unpaired *t* test).

In order to dissect how A20-deficient mast cells exacerbate pathological immune responses *in vivo*, we first addressed IgE/FcεRI-induced mast cell activation that prominently provokes degranulation, in addition to pro-inflammatory NF-κB activation [20]. To explore consequences of A20 deficiency in connective tissue-type mast cells on anaphylactic responses *in vivo*, we performed FcεRI-mediated immediate phase passive cutaneous anaphylaxis (PCA) and passive systemic anaphylaxis (PSA) experiments, which depend on mast cell degranulation [8],[20]. The vascular leakage in IgE primed ears in immediate phase PCA reactions (Figure 7A), as well as changes in core body and skin temperature during PSA reactions (Figure 7B and Figure S5A), did not differ significantly between control and *Mcpt5Cre A20^F/F^* mice. However, we observed a minor trend towards stronger responses in *Mcpt5Cre A20^F/F^* mice. Therefore, we embarked on extensive *in vitro* degranulation analyses using both BMMCs and PMCs. IgE/FcεRI engagement by antigen induced the same extent of degranulation in A20-deficient BMMCs (Figure 7C and D) and PMCs (Figure 7E) in comparison to controls. Prolonged priming with IgE and stimulation with IL-33 or TLR ligands alone or in addition to antigenic challenge did not reveal a role for A20 during degranulation and histamine release (Figure S5B–F). Therefore, the minor increase in *in vivo* responses might be due to the slightly elevated mast cell numbers in *Mcpt5Cre A20^F/F^* in comparison to control mice. Collectively, our results show that A20 does not regulate degranulation and hence anaphylactic responses.

We next performed late phase PCA responses, which are thought to be promoted by NF-κB activation in mast cells [20]. In this model, *Mcpt5Cre A20^F/F^* mice showed significantly enhanced ear swelling compared to controls (Figure 7F and Figure S5G).

Our data thus demonstrate that A20 does not regulate IgE/FcεRI-induced signals leading to degranulation and immediate anaphylactic events but rather late phase reactions.

### A20 Deficiency Causes Mast Cell Hyperactivity Leading to Increased Cytokine Production, Activation Marker Expression, and Alarmin Production in Fibroblasts

**Figure 8:**
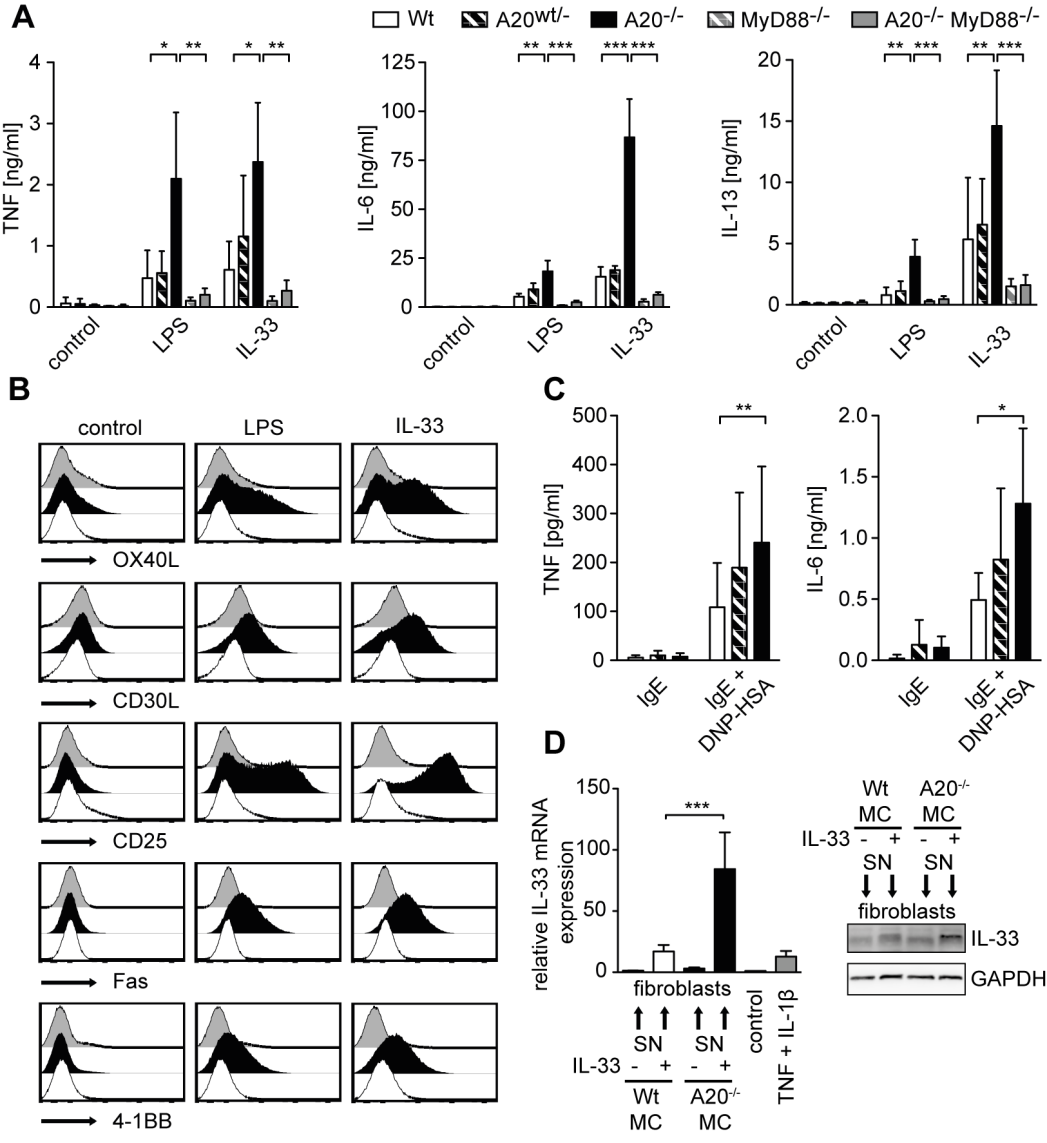
A20 is a key negative regulator of mast cell activation. (A) TNF, IL-6, and IL-13 secretion of BMMCs stimulated for 6 h with 10 µg/mL LPS or 10 ng/mL IL-33 were measured by ELISA. Data are means + SD from three independent experiments. (B) Expression levels of cell surface activation markers on BMMCs stimulated for 3 d with 10 µg/mL LPS or 10 ng/mL IL-33. Histograms are representative of three independent experiments. (C) TNF and IL-6 secretion of BMMCs loaded for 2 h with 1 µg/mL anti-DNP IgE and subsequently stimulated for 6 h with 10 ng/mL DNP–HSA were measured by ELISA. Data are means + SD from at least four independent experiments. (D) Synovial fibroblasts were stimulated with filtered supernatants (SNs) from A20-deficient and control BMMCs, which were activated overnight with 10 ng/mL IL-33. IL-33 mRNA levels in synovial fibroblasts (8 h stimulation with SNs or 10 ng/mL IL-1β and TNF) were determined by quantitative RT-PCR, normalized to PGBD, and changes relative to unstimulated fibroblasts are shown. IL-33 protein levels (24 h stimulation with SNs) were analyzed by Western blotting. Data are means + SD from at least three (RT-PCR) or representative of two (Western blot) independent experiments using SNs from two independent mast cell preparations. *p<0.05, **p<0.01, ***p<0.001 (one-way repeated measures ANOVA).

As A20 deficiency did not affect mast cell degranulation, we examined if enhanced pro-inflammatory reactions could account for the exacerbated responses observed in *Mcpt5Cre A20^F/F^* mice. Hence, we dissected the activation state of A20-deficient mast cells *in vitro*. Stimulation with LPS, IL-33, and IL-1β induced dramatically augmented transcription and secretion of pro-inflammatory cytokines such as TNF, IL-6, and IL-13 from A20-deficient in comparison to control mast cells (Figure 8A and Figure S6A–C). PMA/Iono-induced release of IL-2 was unchanged (Figure S6D). A trend towards an increase in IL-1β secretion was also observed (Figure S6E). In addition, the enhanced responses of A20-deficient mast cells to LPS and IL-33 were demonstrated by increased up-regulation of the activation markers OX40L, CD30L, CD25, Fas, and 4-1BB (Figure 8B). Ablation of MyD88 completely abolished the enhanced production of cytokines and rescued the hyperactive phenotype caused by A20 deficiency (Figure 8A and B and Figure S6B,C, and E). A20-deficient mast cells also produced more pro-inflammatory cytokines than wild-type mast cells in response to priming with IgE and to FcεRI cross-linking (Figure 8C and Figure S6F and G). A recent study proposed that during arthritis IL-33 released by synovial fibroblasts activates mast cells, which in turn, by their secretion of pro-inflammatory cytokines, could enhance IL-33 expression in the former, leading to a paracrine feed-forward loop [38]. Supernatants of IL-33–stimulated A20-deficient hyperactive mast cells induced significantly more IL-33 production in synovial fibroblasts than supernatants of IL-33–stimulated control mast cells (Figure 8D). This indicates that the lack of A20 in mast cells could amplify local IL-33–mediated feed-forward loops to exacerbate inflammation.

Collectively, our results demonstrate that in mast cells A20 is a selective negative feedback regulator of NF-κB–mediated inflammatory signaling events but not of anaphylactic IgE:FcεRI–induced degranulation. Loss of A20 dramatically enhances their pro-inflammatory properties and causes profound mast cell hyperactivation. A20-deficient hyperactive connective tissue-type mast cells exacerbate lung as well as late phase skin inflammation and CIA, pointing to an important contribution of mast cells in these diseases.

## Discussion

Experiments in mast cell-deficient mice that carry hypomorphic c-Kit mutations have not only positioned mast cells as central mediators of allergic and anaphylactic responses but also in inflammatory and autoimmune diseases. However, recent experiments employing novel Kit-independent mast cell-deficient mice challenged this notion, which urged for a reassessment of their exact role in immunological and inflammatory reactions [5]. To study their contribution to particular immune reactions, one can assay the presence of mast cells and their activation status, the effects of mast cell deficiency, or the effects of mast cell hyperactivity, akin to the modified Koch's postulates for immunology [40]. Hence, we established a novel gain-of-function *in vivo* approach by ablating the NF-κB negative feedback regulator A20 specifically in connective tissue-type mast cells. This led to hyperactive mast cells with overshooting inflammatory responses to common disease-causing stimuli. We cannot completely exclude the possibility that loss of A20 has consequences on mast cell differentiation and/or functions in addition to amplifying inflammatory signaling and that this might influence the outcome of our experiments. However, A20's function as a very potent activation-induced negative feedback regulator in mast cells indicates that our interpretation of its role in the context of inflammatory diseases is warranted. This view is strongly supported by the fact that many of the phenotypes we observed can be neutralized by co-ablation of MyD88.

Our results demonstrate that A20 does not regulate instant degranulation, which is triggered by antigen binding to the IgE:FcεRI module and is critical for immediate cutaneous and systemic anaphylactic reactions. In contrast, A20 serves as a feedback inhibitor of FcεRI-initiated NF-κB activity and in its absence the secretion of pro-inflammatory cytokines, and thereby late phase PCA reactions are strongly amplified. Therefore, it should be possible to modulate A20 function without provoking anaphylactic reactions. Although pharmacological augmentation of A20 function is mainly discussed in the context of treating inflammatory and autoimmune diseases [17], its inhibition might be beneficial to enhance immunogenicity of vaccines and the management of viral infections [14],[41].

In addition, also in mast cells A20 limits pro-inflammatory gene expression upon stimulation with TLR ligands and cytokines. Furthermore, inflammation can be triggered by alarmins released by dying cells, such as the recently identified IL-33 [42]. Mast cells can sense IL-33 by virtue of their characteristic high constitutive IL-33R α-chain expression [43]. We discovered that A20 acts as a key negative feedback inhibitor of IL-33–induced MyD88-dependent NF-κB but not MAPK activation in mast cells. A20 deficiency leads to dramatically enhanced cytokine production, activation marker expression, resistance to apoptosis, and increased proliferation upon stimulation with IL-33. As IL-33 can also be sensed by dendritic and myeloid cells [44], IL-33–induced activation in the steady state could contribute to the spontaneous MyD88-dependent pathologies observed in mice that lack A20 specifically in these cell types [12],[14]. Furthermore, IL-33 triggers activation of group 2 innate lymphoid cells, Th2 cells, NKT cells, B cells, NK cells, eosinophils, and basophils [44]. We therefore propose that A20's role in limiting IL-33–mediated NF-κB activation is of general importance in the immune system.

Loss of A20 in connective tissue-type mast cells *in vivo* did not cause dramatic spontaneous inflammation. However, A20-deficient peritoneal mast cells displayed a pre-activated or poised state, which depended entirely on the presence of MyD88. This indicates that mast cells, similar to other innate leukocytes [12],[14], constantly receive tonic signals that are controlled by A20, via one or more MyD88-dependent receptors, such as TLR, IL-1R, IL-18R, or IL-33R. The fact that A20-deficient connective tissue-type mast cells, unlike macrophages or DCs lacking A20 [12]–[14], do not cause pronounced spontaneous inflammation suggests that these cells are intrinsically less potent inducers of inflammatory reactions and/or have developed additional control mechanisms.

*A20* is a susceptibility gene locus for rheumatoid arthritis [17],[18], and mast cells are implicated in the pathogenesis of this disease [34]. Several recent studies pointed to a crucial role of IL-33 during arthritis [45], which could promote joint inflammation, at least in part, by activating mast cells [38]. This notion is in line with our observation that hyperactive A20-deficient mast cells caused earlier onset as well as exacerbation of CIA symptoms. In contrast, A20-deficient connective tissue-type mast cells did not worsen symptoms of MOG peptide-induced EAE, possibly because initiation of this disease is IL-33 independent [33]. Our results do not strictly exclude a role of mast cells in this model, as it remains possible that general mast cell hyperactivation might have different or more pronounced effects than those elicited by the loss of A20 in connective tissue-type mast cells alone. Nevertheless, our results illustrate that profound hyperactivation of the inflammatory properties of connective tissue-type mast cells does not affect the outcome of EAE induction.

Arthritis can develop in absence of an adaptive immune system, driven solely by A20-deficient innate immune cells [12]. We propose that in the context of autoimmune arthritis, A20-deficient mast cells exacerbate local inflammation in the joint. Tissue damage or physical stress during the early stages of arthritis development cause local release of endogenous TLR ligands or alarmins such as IL-33. Pro-inflammatory cytokine secretion by A20-deficient mast cells results in a locally restricted auto-amplification loop by enhancing IL-33 expression in synovial fibroblasts leading to stronger mast cell activation in a paracrine fashion. This scenario is supported by our data that MyD88 deficiency in mast cells counteracts the early onset and disease severity caused by ablation of A20.

Amplification of detrimental IL-33–mediated feed-forward loops by loss of A20 function might not be restricted to initial stages in arthritis but could also play an important role in other inflammatory conditions including psoriasis, systemic sclerosis, inflammatory bowel disease, and asthma, which have an IL-33 component and are associated with *A20* or A20 binding partner (*TNIP1*) gene locus polymorphisms [17]–[19],[44],[45]. Furthermore, IL-33 is increased in human asthmatics, and recent genome-wide association studies have identified the genes encoding for IL-33 and its receptor as susceptibility loci for asthma [44]. Also in our asthma models, IL-33 is released by stromal cells, such as epithelial and smooth muscle cells, after HDM provocation [28],[46]. Mast cell numbers increase during allergic airway inflammation and then localize within the smooth muscle, where they enhance bronchial hyperreactivity [47]. This localization hence enables mast cells to easily sense IL-33, and A20 deficiency amplifies their ensuing responses, exacerbating immune cell activation and lung inflammation. Fittingly, IL-33 could also be used as an adjuvant to induce Th2 immunity, which was enhanced by mast cells in *Mcpt5Cre A20^F/F^* mice, possibly by promoting an initial pro Th2 innate response to IL-33 as indicated by the increased DC numbers in the airways. The stronger general immune activation elicited by alum probably overrides these mast cell-dependent effects in the OVA/alum model [26],[27]. Since the HDM model is much closer to the human situation, our data clearly indicate that inflammatory functions of connective tissue-type mast cells can contribute to allergic inflammatory airway diseases. The role of mast cells in airway hyperreactivity has been studied predominantly in mast cell-deficient c-Kit mutant mouse strains, which did not allow dissecting the role of individual mast cell subsets [26],[27]. Our model, with selective hyperinflammatory properties of connective tissue-type mast cells, points to an important role for this subset in controlling asthma. However, our findings do exclude a role for mucosal mast cells in this disease.

In conclusion, our data demonstrate that loss of A20 specifically amplifies NF-κB controlled gene expression programs in connective tissue-type mast cells during inflammatory, allergic, and autoimmune conditions. We demonstrate that, in addition to its known function downstream of TLRs and the IL-1R, A20 also plays a critical role as a negative feedback inhibitor of IL-33R– and FcεRI-initiated pro-inflammatory signaling pathways. It does not, however, regulate IgE-dependent anaphylactic responses. As a result, the magnitude of inflammatory responses can be controlled through pharmacological intervention modulating A20 levels or activity without affecting anaphylactic reactions. Our study demonstrates, to our knowledge for the first time, the consequences of inflammatory mast cell hyperreactivity, identifying mast cells as therapeutic targets in airway inflammation and autoimmune arthritis. Our data also suggest that alterations in mast cell function could contribute to the pathologies linked to genetic polymorphisms in the *A20* gene locus or some of its binding partners (for example, TNIP1) that are associated with autoimmune, such as rheumatoid arthritis, or allergic diseases [17]–[19].

## Materials and Methods

### Genetically Modified Mice

*Mcpt5Cre* [23], *A20^F^* [16], *MyD88^F^* [24], *R26-Stop^F^YFP* [48], and *Kit^CreERT2^* mice [21],[22] were kept on a C57BL/6 genetic background. All animal procedures were approved by the Regierung of Oberbayern and the Animal Ethics Committee of the University of Ghent.

### Passive Cutaneous and Systemic Anaphylaxis

For immediate phase PCA reactions, mice were passively sensitized by intradermal injection of 100 ng anti-DNP IgE (SPE-7 supernatant) into one ear and PBS into the contralateral ear. After 24 h, mice were challenged by intravenous (i.v.) injection of 200 µg DNP-HSA in 0.5% Evans blue (both Sigma-Aldrich). Extravasation was quantified by dimethylformamid extraction and photometric quantification.

For late phase PCA reactions, mice were sensitized by i.v. injection of 20 µg anti-DNP IgE (SPE-7 supernatant) and 24 h later challenged by epicutaneous application of 20 µL 0.2% DNFB (Sigma-Aldrich) in acetone/olive oil (4∶1) to one ear and vehicle to the contralateral ear followed by measuring ear thickness over time using a thickness gauge (Mitutoyo).

For PSA reactions, mice were sensitized by intraperitoneal (i.p.) injection of 10 µg anti-DNP IgE (SPE-7 supernatant) and 24 h later challenged by i.p. injection of 100 µg DNP-HSA. Systemic anaphylactic response was monitored by measuring changes in body temperature using a rectal thermometer (Bioseb) or changes of dorsal skin temperature using a thermography camera (Jenoptik).

### Asthma Models

OVA-specific allergic airway inflammation was induced in 6–10-wk-old mice, either by immunization with i.p. injections of 10 µg OVA on 7 alternated days or by an i.p. injection of OVA/Alum (10 µg OVA adsorbed to 1 mg aluminum hydroxide; Sigma-Aldrich) at day 0 and day 7. In both models, mice were challenged 10 d after the last i.p. OVA injection with OVA aerosols delivered from a jet nebulizer delivering 1% OVA in PBS for 30 min/day for 3 consecutive days. Twenty-four hours after the last challenge, BAL fluid, blood, and the MLNs were collected. IL-13 was measured in the supernatant of 10^6^ MLN cells restimulated with 15 µg/mL OVA for 4 d.

To induce HDM-specific allergic airway inflammation, mice were sensitized intranasally (i.n.) with 1 µg HDM extracts (Greer Laboratories) on day 0 under isoflurane sedation and were subsequently challenged with 10 µg HDM i.n. on days 7–11. On day 15, allergic airway inflammation was characterized as described above. To assay DC responses, mice received 100 µg HDM extracts (Greer Laboratories) and 10 µg OVA-AF647 (Invitrogen) intratracheally under isoflurane sedation and were analyzed 18 h later.

To address the innate immune response to IL-33, mice were treated on 3 consecutive days with 100 ng IL-33 i.n., and lungs were harvested on day 4.

To provoke IL-33–induced airway inflammation, mice were sensitized with 25 ng IL-33 (BioLegend) and 10 µg OVA and boosted with 10 µg OVA day 7. OVA aerosols challenges were given from day 17 to 19, and at day 20 airway inflammation was assessed as described above.

### EAE

Mice were immunized subcutaneously at two sites with 200 µg MOG_35–55_ emulsified in Freund's adjuvant (Sigma-Aldrich) supplemented with 5 mg/mL *Mycobacterium tuberculosis* (strain H37Ra, Difco). On the day of and 2 d after immunization, 400 ng pertussis toxin (List Biological Laboratories) was administered i.p. EAE severity was assessed every day based on a clinical scoring system [49]: 0, normal; 0.5, partial limp tail; 1, complete paralysis of the tail; 2, loss in coordinated movement, hind limb weakness; 2.5, one hind limb paralyzed; 3, both hind limbs paralyzed; 4, forelimbs paralyzed; and 5, moribund or dead.

### CIA

Male mice were immunized intradermally at two sites at the base of the tail with 200 µg chicken type II collagen in Freund's Adjuvant containing 1 mg/mL *Mycobacterium tuberculosis* (Sigma-Aldrich) essentially as described [50]. Arthritis severity was assessed every second day based on a clinical scoring system: 0, normal; 1, slight swelling and/or erythema; 2, pronounced edematous swelling; and 3, ankylosis. Each limb was graded, giving a maximum score of 12. Paw thickness was measured every second day using a thickness gauge (Mitutoyo). For histology hind paws were fixed in 4% PFA, decalcified in 10% buffered EDTA, embedded in paraffin, sectioned, and stained with hematoxylin and eosin.

### Flow Cytometry

Nonspecific binding of antibodies to isolated single cells was minimized by Fc-block (CD16/32, 93, eBioscience; 2.4G2, BD), and cells were stained with monoclonal antibodies against 4-1BB (17B5), B220 (RA3-6B2), CD3ε (145-2C11) CD4 (RM4-5), CD8 (53-6.7), CD11b (M1/70), CD11c (N418), CD19 (1D3), CD25 (PC61.5), CD30L (RM153), CD44 (IM7), CD45.2 (104), CD62L (MEL-14), CD80 (16-10A1), CD86 (GL1), c-Kit (2B8), FcεRI (MAR-1), Gr-1 (RB6-8C5), Ly6C (HK1.4), MHC Class I (AF6-88.5.5.3), MHC Class II (M5/114.15.2), OX40L (RM134L), TCRβ (H57-597) (all eBioscience), Fas (Jo2), Ly6G (1A8), Siglec-F (E50-2440) (all BD), and ST2 (DJ8, MD Bioproducts). Biotinylated antibodies were detected with fluorophore-conjugated streptavidin (eBioscience).

Cytokine secretion of peritoneal cells was blocked for 4 h with monensin (eBioscience) in the absence of stimulation. Cells were fixed with 2% paraformaldehyde (PFA), permeabilized with 1% saponin (Sigma-Aldrich), and stained with monoclonal antibodies against IL-4 (BVD-24G2), IL-13 (eBio13A), and TNF (MP6-XT22) (all eBioscience).

Dead cells were excluded using 7-AAD (eBioscience) or EMA (Invitrogen). Samples were acquired on FACSCalibur and FACSCantoII or sorted on FACSAriaII (BD) machines, and analyzed with FlowJo software (Treestar).

To initiate growth factor deprivation-induced apoptosis, BMMCs were extensively washed with PBS and cultured in simple mast cell medium lacking IL-3 and SCF or supplemented with 5 µg/mL anti-DNP IgE, 10 µg/mL LPS (both Sigma-Aldrich), or 10 ng/mL IL-33 (PeproTech). Cell death was quantified at the indicated time points after propidium iodide staining (10 µg/mL; Sigma-Aldrich) and flow cytometric analysis.

Cell cycle analysis was conducted by staining EtOH-fixed BMMCs treated with 100 µg/mL RNase A using 50 µg/mL propidium iodide (both Sigma-Aldrich).

Flow cytometric analysis of BAL fluid composition was performed according to a recently described method [29].

To prepare skin single-cell suspensions, dissected ears were digested in DMEM (Gibco) containing 25 mM Hepes (PAN), 0.05 mg/mL Liberase TM, 0.2 mg/mL DNase I (both Roche Diagnostics), and 0.5 mg/mL Hyaluronidase (Sigma-Aldrich) at 37°C for 1 h and grinded through a 70 µm cell strainer (BD).

To prepare lung and MLN single-cell suspensions, lungs and MLNs were digested using RPMI containing 0.02 mg/mL Liberase TM and 10 U DNase (both Roche Diagnostics).

### Immunofluoresence and Immunohistology

For whole-mount ear skin immunohistology, ears were separated into dorsal and ventral sheets, and cartilage-free ear sheets were fixed by floating on 1% PFA overnight at 4°C. For back skin immunohistology, frozen 12 µm sections were thawed, air dried, and fixed with methanol for 10 min at −20°C. Ear sheets and back skin sections were blocked with 1% BSA and stained with a rabbit anti-laminin antibody (gift from Michael Sixt) followed by Cy3-conjugated anti-rabbit (Jackson ImmunoResearch), and FITC-conjugated avidin (Zymed). Images were acquired with a fluorescent microscope (Zeiss AxioImager Z1).

To study vascular permeability in the airways, mice were sensitized (day 0; 1 µg/mouse, intratracheally (i.t.)) and challenged (day 7; 10 µg/mouse; i.t.) with HDM. One hour after the i.t. application, mice were injected intravenously with FITC^+^ 500 nm microbeads (Invitrogen). Five minutes later mice were sacrificed, blood was removed by perfusion with PBS followed by PFA, and the trachea was isolated and cleaned. Blood vessels were visualized by subsequent incubation of the trachea with 5% normal goat serum in PBS with 3% Triton X-100 (PBS plus) (1 h, RT), rat anti-mouse CD31 (1/500 in PBS plus, overnight, 4°C), and AF647 coupled goat anti-rat IgG (1/500 in PBS plus, 4 h, RT), separated by several washes with PBS plus. Trachea whole mounts were embedded in polyvinyl alcohol mounting medium (DABCO Fluca). Beads and blood vessels were visualized by confocal microscopy (Zeiss LSM 710).

### Cell Culture

To generate BMMCs and PMCs, bulk bone marrow or peritoneal cells were cultured in suspension in mast cell medium: DMEM (Gibco) supplemented with 10% FCS (PAA), 2% supernatant from X63/0 cells expressing IL-3 (gift from Ton Rolink), 0.5% supernatant from CHO cells expressing SCF (gift from Patrice Dubreuil), Glutamax (Gibco), Non Essential Amino Acids (Gibco), 50 µM 2-mercaptoethanol (Merck), and 25 mM Hepes (PAN). After 4 wk, BMMCs were cultured in mast cell medium containing 1 µM 4-hydroxytamoxifen (Sigma-Aldrich) for 7 d to delete conditional alleles *in vitro* (BMMCs, *Kit^CreERT2/+^* = *Wt*; *Kit^CreERT2/+^A20^+/F^* = *A20^wt/^* ^−^; *Kit^CreERT2/+^A20^F/F^* = *A20* ^−/−^; *Kit^CreERT2/+^MyD88^F/F^* = *MyD88* ^−/−^; *Kit^CreERT2/+^A20^F/F^MyD88^F/F^* = *A20* ^−/−^ *MyD88* ^−/−^; PMCs, *Mcpt5Cre* = *Wt*; *Mcpt5Cre A20^F/F^* = *A20* ^−/−^; *Mcpt5Cre A20^F/F^MyD88^F/F^* = *A20* ^−/−^ *MyD88* ^−/−^). Purity was checked based on the expression of FcεRI and c-Kit by flow cytometric analysis.

Murine synovial fibroblasts were isolated and cultured as described previously [51] with slight modifications. In brief, ankle joints were dissected, separated by cutting through the joint space, and digested in DMEM (Gibco) containing 25 mM Hepes (PAN), 0.05 mg/mL Liberase TM, and 0.2 mg/mL DNase I (both Roche Diagnostics) at 37°C for 1 h.

### Signal Transduction and Western Blot

Whole cell lysates were prepared by lysing cells for 30 min on ice in RIPA buffer (50 mM Tris/HCl pH 7.4, 150 mM NaCl, 1% NP40, 1 mM EDTA, 0.25% Na-deoxycholate) supplemented with 10 µg/mL Aprotinin, 10 µg/mL Leupeptin, 0.1 mM Na_3_VO_4_, 1 mM PMSF, 10 mM NaF, 1 mM DTT, and 8 mM β-Glycerophosphate after stimulation with LPS (Sigma-Aldrich), TNFα, IL-1β, IL-33 (all PeproTech), or FcεRI cross-linking (anti-DNP IgE, SPE-7 supernatants; DNP-HSA, Sigma-Aldrich). PVDF membranes were blotted with the following antibodies: A20, I-κBα (both Santa Cruz), Bcl-x_L_ (BD), AKT, phospho-AKT, ERK, phospho-ERK, phospho-I-κBα, JNK, phospho-JNK, p38, phospho-p38 (all Cell Signaling), MyD88 (Stressgen), IL-33 (Enzo Life Sciences), and GAPDH (Calbiochem).

### ELISA

TNF, IL-13, IL-1β (all PeproTech), IL-6, and IL-2 (both BD) levels were determined by ELISA as recommended by the manufacturer.

### Real-Time PCR

RNA was isolated (USB) and reverse transcribed (Promega) for quantitative real-time PCR using TaqMan Gene Expression Assays (A1, Applied Biosystems) or Universal Probe Library (all other genes, Roche Diagnostics) probes and primers as recommended by the manufacturer.

DNA from sorted cells was isolated (Life Technologies), and quantitative real-time PCR (10 min 95°C, 50 cycles: 10 s 95°C, 30 s 60°C, 1 s 72°C) was performed on DNA corresponding to 10,000 cells in a reaction volume of 20 µL containing 1× TaqMan Probe Master (Roche Diagnostics), 1 µM of each forward and reverse primers (A20 locus a, 5′-ACTGTTTGAAGCATGCACGA-3′; b, 5′-ACAACCTGTCAAATCCATATTCAG-3′; A20 deleted c, 5′-AAATCTGGACAGCTGATTCCT-3′; d, 5′-CAACATCTCAGAAGGACACCAT-3′) and 0.1 µM TaqMan probe #68 (A, Roche Diagnostics) or loxP probe (B, 5′-6-FAM-atAaCtTCgtatagCATaCattatac-BHQ-1-3′; capital letters = LNA; Eurogentec). In order to evaluate sensitivity of the real-time PCR, we generated samples containing 1,000 (10%), 100 (1%), or 10 (0.1%) A20^−/−^ BMMCs among 9,000, 9,900, or 9,990 A20^F/F^ splenocytes.

### Mast Cell Degranulation

To induce degranulation, BMMCs and PMCs were loaded for 2 or 24 h with 1 µg/mL anti-DNP IgE (SPE-7 supernatant). After washing in Tyrode's buffer (10 mM Hepes, pH 7.3, 135 mM NaCl, 5 mM KCl, 1.8 mM CaCl_2_, 1 mM MgCl_2_, 5.6 mM glucose, 0.5 mg/mL BSA), cells were stimulated with the concentrations of DNP–HSA indicated in the figure, 10 ng/mL IL-33, 10 µg/mL LPS, or 500 ng/mL A23187 for 30 min. β-hexosaminidase activity in supernatants and cell pellets solubilized with 0.5% Triton X-100 in Tyrode's buffer were measured with p-nitrophenyl-N-acetyl-β-D-glucosaminide (Sigma-Aldrich). To measure degranulation using Annexin V binding, after 20 min of FcεRI cross-linking, BMMCs were washed in Annexin V binding buffer (25 mM Hepes, pH 7.2, 140 mM NaCl, 2.5 mM CaCl_2_), and Cy3-conjugated Annexin V (gift from Dirk Mielenz) binding was analyzed by flow cytometric analysis. To measure histamine release, PMCs were loaded 24 h with 1 µg/mL anti-DNP IgE (SPE-7 supernatant), stimulated with the concentrations of DNP–HSA indicated in the figure, 10 ng/mL IL-33, 10 µg/mL LPS, or 500 ng/mL A23187 for 30 min, and histamine levels were determined by EIA as recommended by the manufacturer (Immunotech).

### Statistics

Statistical analysis of the results was performed by Log-rank (Mantel-Cox) test, Mann-Whitney U test, Student's *t* test, or by one-way ANOVA followed by Tukey's test. The *p* values are presented in figure legends where a statistically significant difference was found.

## Supporting Information

Figure S1 **A20 expression is induced by IL-1β, LPS, IL-33, and FcεRI cross-linking in mast cells.** (A) Wild-type and A20-deficient BMMCs were stimulated with 10 ng/mL IL-1β for 3 h. A20 mRNA levels were determined by quantitative RT-PCR, and protein levels were assessed by Western blotting. Changes in transcript levels relative to unstimulated cells are shown after normalization to PBGD. Data are means + SD (RT-PCR) from three or representative (Western blot) of three independent experiments. (B) Wild-type and A20-deficient BMMCs were stimulated with 10 µg/mL LPS or 10 ng/mL IL-33 for the indicated time intervals. To induce FcεRI cross-linking, BMMCs were loaded overnight with 1 µg/mL anti-DNP IgE and subsequently stimulated for the indicated time intervals with 10 ng/mL DNP–HSA. A20 protein levels were assessed by Western blotting and are representative of three independent experiments. Quantifications are shown in Figure 1A. (C) Representative dot plots showing FcεRI and c-Kit expression on BMMCs of the indicated genotypes and proportions of FcεRI^+^ and c-Kit^+^ cells. (D) Changes in phosphorylated protein normalized to nonphosphorylated protein levels and I-κBα levels normalized to GAPDH relative to unstimulated wild-type BMMCs at time point 0 h are shown. Data are geometric means from at least two independent experiments.
(TIF)

Figure S2 **Mild cellular expansions in mast cell-specific A20-deficient mice.** (A) Representative immunofluorescence images of dorsal skin sections: green, avidin-FITC; red, anti-laminin; blue, DAPI; scale bar, 100 µm. Scatter plot shows mast cell frequencies in dorsal skin sections. Individual data points represent mean mast cell numbers in 10 fields of view per mouse. Bars indicate means from at least six mice per genotype (Control, 7 *Mcpt5Cre* mice). (B) Dot plots showing proportions of cytokine positive ex vivo isolated peritoneal mast cells (c-Kit^+^). Numbers represent means ± SD from at least eight mice per genotype (Control, 9 *Mcpt5Cre* and 2 Cre^−^ littermates). (C) Western blot analysis of A20 and MyD88 protein levels in PMCs of the indicated genotypes. Data are representative of five independent mast cell preparations (Control, 4 *Mcpt5Cre* and 1 Cre^−^ littermate). (D) Schematic representation of the A20 conditional allele before and after Cre-mediated recombination (open squares, exons; closed triangles, loxP sites) and location of real-time PCR primers (a, b, A20 locus; c, d, deleted A20 locus) and probes (A, A20 locus; B, deleted A20 locus). Ratios of genomic DNA corresponding to the deleted A20 locus relative to the A20 locus (ratio (deleted:A20 locus) = 2^Cp(A20 locus)-Cp(deleted)^) were determined by quantitative real-time PCR using locus-specific primers and fluorescent-labeled TaqMan probes. Samples containing 10%, 1%, or 0.1% A20^−/−^ BMMCs among 90%, 99%, or 99.9% A20^F/F^ splenocytes were used to determine the detection limit. Splenic T cells (TCRβ^+^B220^−^), B cells (TCRβ^−^B220^+^), DCs (CD11c^high^), eosinophils (eos, CD11c^−^CD11b^+^SiglecF^+^SSC-A^high^), monocytes/macrophages (monos/macs, CD11c^−^CD11b^+^SiglecF^−^Gr-1^int^), neutrophils (neutros, CD11c^−^CD11b^+^SiglecF^−^Gr-1^high^), and peritoneal cavity macrophages (PC macs, CD11b^high^c-Kit^−^) were sorted from *Mcpt5Cre A20^F/F^* mice. Bars represent means + SD from three mice (splenic subsets) or two mice (PC macs). (E) Pictures of representative spleens from mice of the indicated genotypes. Scatter plot shows absolute splenocyte numbers. Bars are means from at least 13 mice per genotype (Control, 8 *Mcpt5Cre* and 5 Cre^−^ littermates).
(TIF)

Figure S3 **IL-33–induced airway inflammation is enhanced in** ***Mcpt5Cre A20^F/F^*** **mice.** Mice were administered 100 ng IL-33 i.n. on 3 consecutive days. Scatter plots show absolute lung cell numbers of Eos (eosinophils, SSC^hi^, Ly6C^+^, Ly6G^int^, CD11b^+^, CD11c^−^), Monos (monocytes, SSC^int^, Ly6C^+^, Ly6G^lo^, CD11b^+^, CD11c^−^), and Neutros (neutrophils, SSC^hi^, Ly6C^+^, Ly6G^hi^, CD11b^+^, CD11c^−^) as identified by flow cytometric analysis. Bars indicate means from three to six mice per group (Control, 3 “PBS” and 5 “IL-33” Cre^−^ littermates). *p<0.05 (Mann-Whitney test).
(TIF)

Figure S4 **Mast cell-specific A20-deficient mice develop exaggerated CIA.** (A) Increase in paw thickness during CIA was measured using an engineer's micrometer. Data are means ± SEM (for visual clarity instead of SD) from at least 10 mice per genotype (Control, 16 *Mcpt5Cre* mice). (B) Histological sections of ankle joints from CIA mice stained with hematoxylin and eosin. (C) Serum TNF levels in CIA mice were measured by ELISA. Bars indicate medians from at least 10 mice per genotype (Control, 13 *Mcpt5Cre* mice). (D) Scatter plots show absolute cell numbers of total splenocytes, B cells (B220^+^), T cells (TCRβ^+^), and CD4^+^ and CD8^+^ T cell (TCRβ^+^) subsets, and bars indicate means from at least five mice per genotype (Control, 5 *Mcpt5Cre* mice) (effector-like, CD44^hi^CD62L^lo^; memory-like, CD44^hi^CD62L^hi^; naive, CD44^lo-int^CD62L^hi^). *p<0.05, **p<0.01 (one-way ANOVA).
(TIF)

Figure S5 **Normal degranulation and immediate but exacerbated late phase anaphylactic responses in the absence of A20.** (A) Data show dorsal skin temperatures over time during PSA reactions measured with a thermography camera and are means ± SD from at least four mice per genotype (Control, 5 Cre^−^ littermates). (B–E) BMMCs (B and C) and PMCs (D and E) were loaded for 24 h with 1 µg/mL anti-DNP IgE and subsequently stimulated for 30 min with the indicated concentrations of DNP–HSA, 10 ng/mL IL-33, 10 µg/mL LPS, or 500 ng/mL A23187. Degranulation was determined by measuring the activity of β-hexosaminidase in supernatants and cell lysates. (F) PMCs were loaded for 24 h with 1 µg/mL anti-DNP IgE and subsequently stimulated for 30 min with the indicated concentrations of DNP–HSA, 10 ng/mL IL-33, 10 µg/mL LPS, or 500 ng/mL A23187. Histamine release was measured by EIA. Two experiments are shown. Data are means + SD from six (B), five (C), five (D), three (E), or three (F) independent mast cell preparations. (G) Histological ear skin sections of late phase PCA reactions stained with hematoxylin and eosin.
(TIF)

Figure S6 **A20 is a key negative regulator of mast cell activation.** (A) BMMCs were stimulated for 6 h with 10 µg/mL LPS or 10 ng/mL IL-33. TNF, IL-6, and IL-13 mRNA levels were determined by quantitative RT-PCR. Changes in transcript levels were calculated after normalization to PBGD. Data are means + SD from four independent experiments. *p<0.05, **p<0.01 (one-way ANOVA). (B) TNF, IL-6, and IL-13 secretion of BMMCs stimulated for 6 h with 10 ng/mL IL-1β were measured by ELISA. Data are means + SD from three independent experiments. *p<0.05, **p<0.01 (one-way repeated measures ANOVA). (C) BMMCs were stimulated as in (B). TNF, IL-6, and IL-13 mRNA levels were determined by quantitative RT-PCR. Changes in transcript levels were calculated after normalization to PBGD. Data are means + SD from three independent experiments. *p<0.05, **p<0.01, ***p<0.001 (one-way ANOVA). (D) BMMCs and PMCs were stimulated with 10 µg/mL LPS, 10 ng/mL IL-33, or 40 nM Phorbol-12-myristate-13-acetate (PMA) and 400 nM Ionomycin (Iono) for 6 h. BMMCs and PMCs were loaded for 2 h with 1 µg/mL anti-DNP IgE and subsequently stimulated for 6 h with 10 ng/mL DNP–HSA. IL-2 secretion was measured by ELISA. Data are means + SD from three independent experiments (BMMCs) or three independent mast cell preparations (PMCs). (E) IL-1β secretion of BMMCs stimulated for 6 h as in (A) were measured by ELISA. Data are means + SD from three independent experiments. (F) IL-13 secretion of BMMCs loaded for 2 h with 1 µg/mL anti-DNP IgE and subsequently stimulated for 6 h with 10 ng/mL DNP–HSA was measured by ELISA. Data are means + SD from three independent experiments. (G) BMMCs were stimulated as in (F). TNF, IL-6, and IL-13 mRNA levels were determined by quantitative RT-PCR. Changes in transcript levels were calculated after normalization to PBGD. Data are means + SD from three independent experiments. *p<0.05, **p<0.01, ***p<0.001 (one-way ANOVA).
(TIF)

## Abbreviations

BAL: bronchoalveolar lavage
BMMCs: bone-marrow-derived mast cells
DCs: dendritic cells
CIA: collagen-induced arthritis
EAE: experimental autoimmune encephalomyelitis
FcεRI: high affinity IgE receptor
HDM: house dust mite
MLN: mediastinal lymph node
PCA: passive cutaneous anaphylaxis
PMCs: peritoneal cavity-derived mast cells
PSA: passive systemic anaphylaxis
