# Supplementary figures and images for: A20-Deficient Mast Cells Exacerbate Inflammatory Responses In Vivo
Source: PLoS Biol. 2014 Jan 14;12(1):e1001762. doi: 10.1371/journal.pbio.1001762 (PMC3891641; doi:10.1371/journal.pbio.1001762)

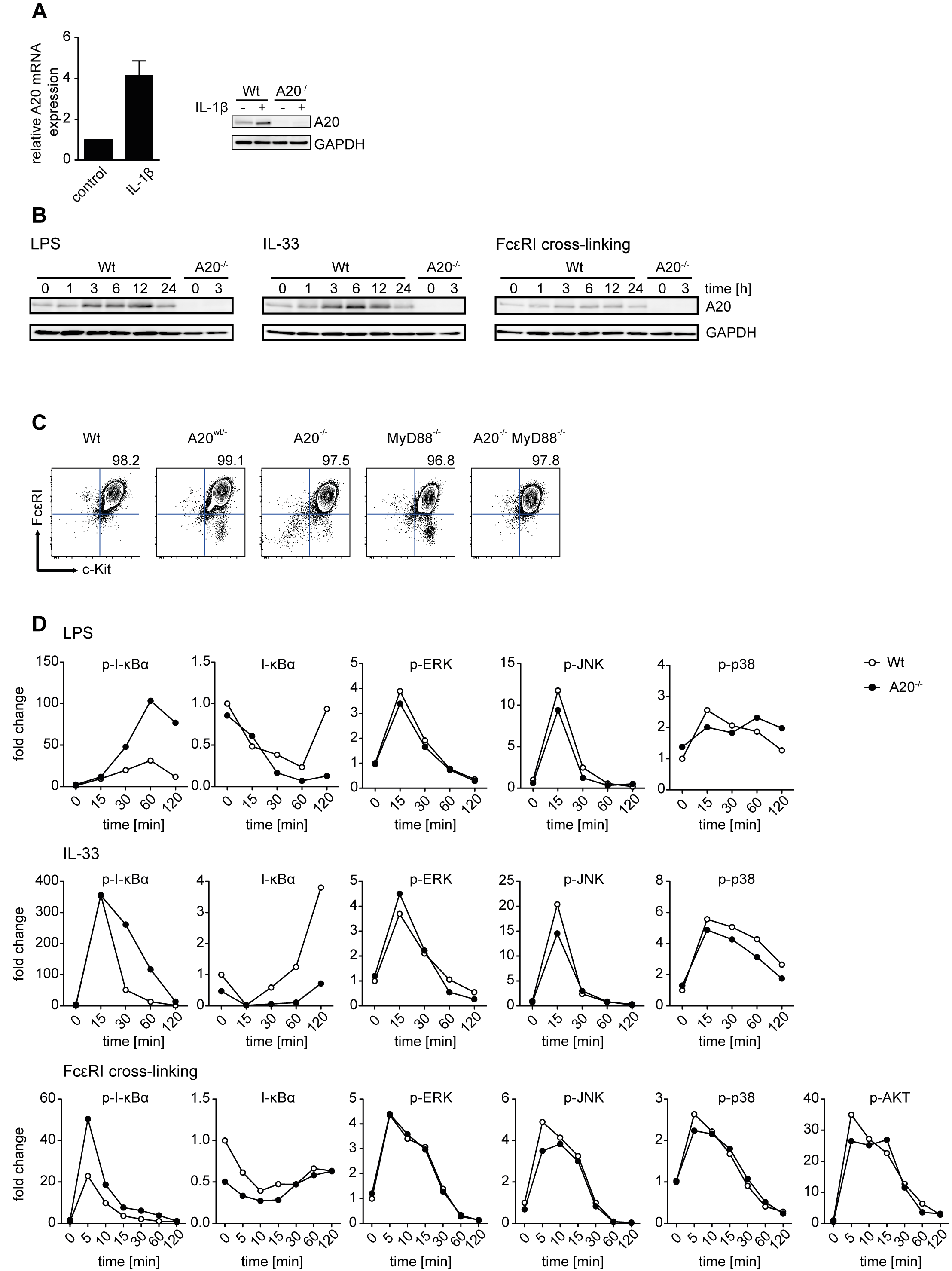

Supplement: Figure S1 — A20 expression is induced by IL-1β, LPS, IL-33, and FcεRI cross-linking in mast cells. (A) Wild-type and A20-deficient BMMCs were stimulated with 10 ng/mL IL-1β for 3 h. A20 mRNA levels were determined by quantitative RT-PCR, and protein levels were assessed by Western blotting. Changes in transcript levels relative to unstimulated cells are shown after normalization to PBGD. Data are means + SD (RT-PCR) from three or representative (Western blot) of three independent experiments. (B) Wild-type and A20-deficient BMMCs were stimulated with 10 µg/mL LPS or 10 ng/mL IL-33 for the indicated time intervals. To induce FcεRI cross-linking, BMMCs were loaded overnight with 1 µg/mL anti-DNP IgE and subsequently stimulated for the indicated time intervals with 10 ng/mL DNP–HSA. A20 protein levels were assessed by Western blotting and are representative of three independent experiments. Quantifications are shown in Figure 1A. (C) Representative dot plots showing FcεRI and c-Kit expression on BMMCs of the indicated genotypes and proportions of FcεRI+ and c-Kit+ cells. (D) Changes in phosphorylated protein normalized to nonphosphorylated protein levels and I-κBα levels normalized to GAPDH relative to unstimulated wild-type BMMCs at time point 0 h are shown. Data are geometric means from at least two independent experiments. (TIF) [file pbio.1001762.s001.tif]

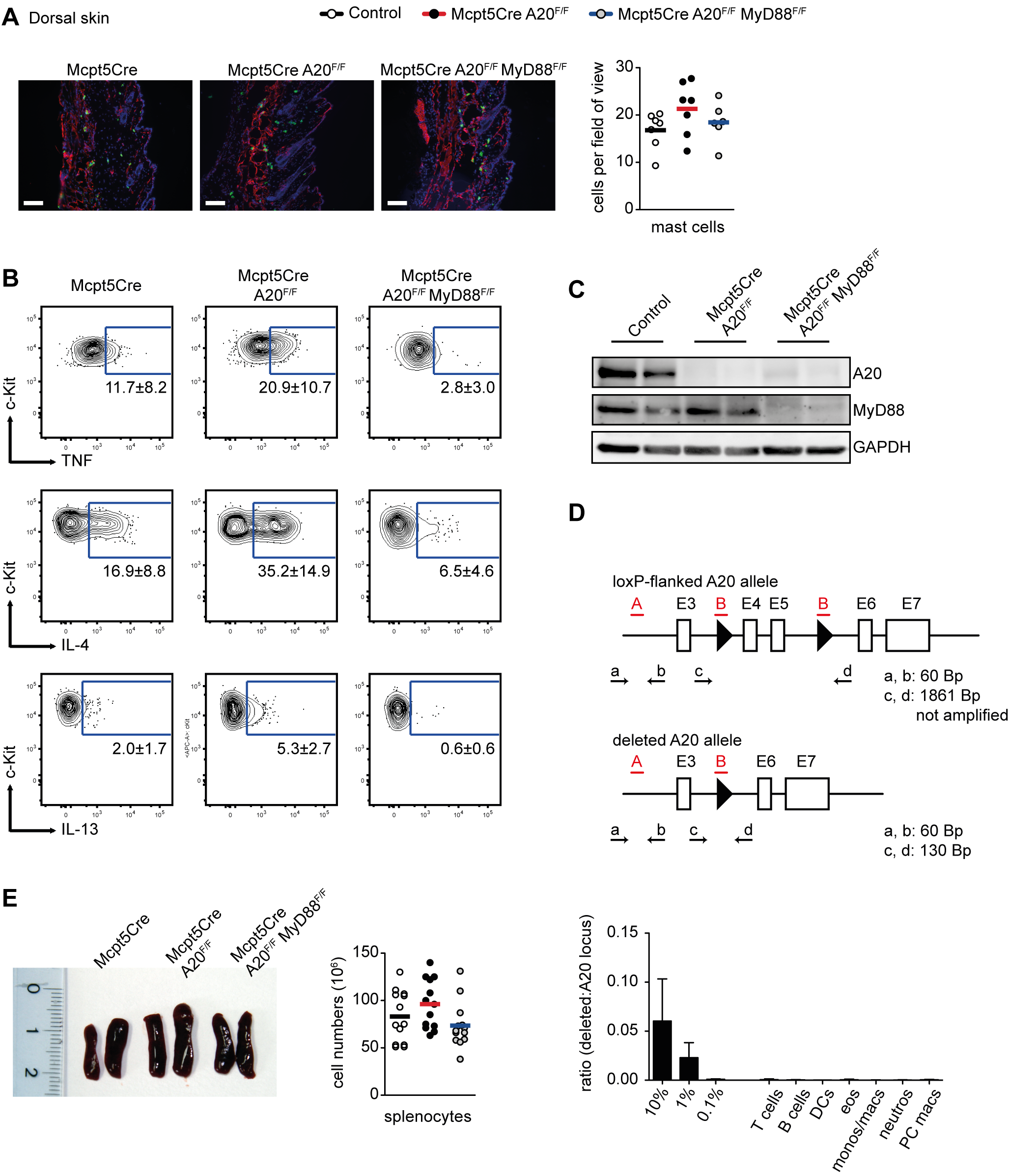

Supplement: Figure S2 — Mild cellular expansions in mast cell-specific A20-deficient mice. (A) Representative immunofluorescence images of dorsal skin sections: green, avidin-FITC; red, anti-laminin; blue, DAPI; scale bar, 100 µm. Scatter plot shows mast cell frequencies in dorsal skin sections. Individual data points represent mean mast cell numbers in 10 fields of view per mouse. Bars indicate means from at least six mice per genotype (Control, 7 Mcpt5Cre mice). (B) Dot plots showing proportions of cytokine positive ex vivo isolated peritoneal mast cells (c-Kit+). Numbers represent means ± SD from at least eight mice per genotype (Control, 9 Mcpt5Cre and 2 Cre− littermates). (C) Western blot analysis of A20 and MyD88 protein levels in PMCs of the indicated genotypes. Data are representative of five independent mast cell preparations (Control, 4 Mcpt5Cre and 1 Cre− littermate). (D) Schematic representation of the A20 conditional allele before and after Cre-mediated recombination (open squares, exons; closed triangles, loxP sites) and location of real-time PCR primers (a, b, A20 locus; c, d, deleted A20 locus) and probes (A, A20 locus; B, deleted A20 locus). Ratios of genomic DNA corresponding to the deleted A20 locus relative to the A20 locus (ratio (deleted:A20 locus) = 2Cp(A20 locus)-Cp(deleted)) were determined by quantitative real-time PCR using locus-specific primers and fluorescent-labeled TaqMan probes. Samples containing 10%, 1%, or 0.1% A20−/− BMMCs among 90%, 99%, or 99.9% A20F/F splenocytes were used to determine the detection limit. Splenic T cells (TCRβ+B220−), B cells (TCRβ−B220+), DCs (CD11chigh), eosinophils (eos, CD11c−CD11b+SiglecF+SSC-Ahigh), monocytes/macrophages (monos/macs, CD11c−CD11b+SiglecF−Gr-1int), neutrophils (neutros, CD11c−CD11b+SiglecF−Gr-1high), and peritoneal cavity macrophages (PC macs, CD11bhighc-Kit−) were sorted from Mcpt5Cre A20F/F mice. Bars represent means + SD from three mice (splenic subsets) or two mice (PC macs). (E) Pictures of representative s [file pbio.1001762.s002.tif]

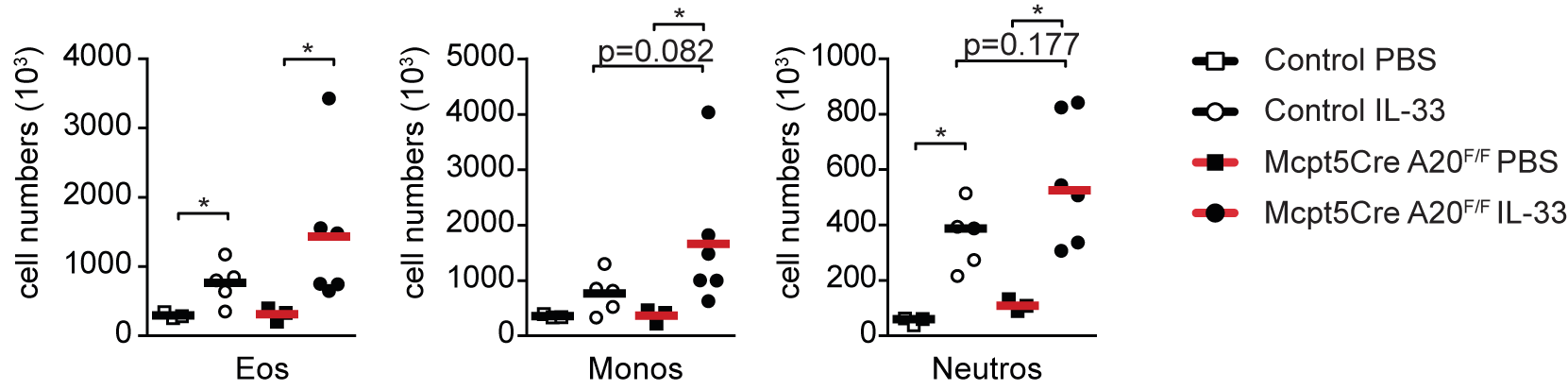

Supplement: Figure S3 — IL-33–induced airway inflammation is enhanced in Mcpt5Cre A20F/F mice. Mice were administered 100 ng IL-33 i.n. on 3 consecutive days. Scatter plots show absolute lung cell numbers of Eos (eosinophils, SSChi, Ly6C+, Ly6Gint, CD11b+, CD11c−), Monos (monocytes, SSCint, Ly6C+, Ly6Glo, CD11b+, CD11c−), and Neutros (neutrophils, SSChi, Ly6C+, Ly6Ghi, CD11b+, CD11c−) as identified by flow cytometric analysis. Bars indicate means from three to six mice per group (Control, 3 “PBS” and 5 “IL-33” Cre− littermates). *p<0.05 (Mann-Whitney test). (TIF) [file pbio.1001762.s003.tif]

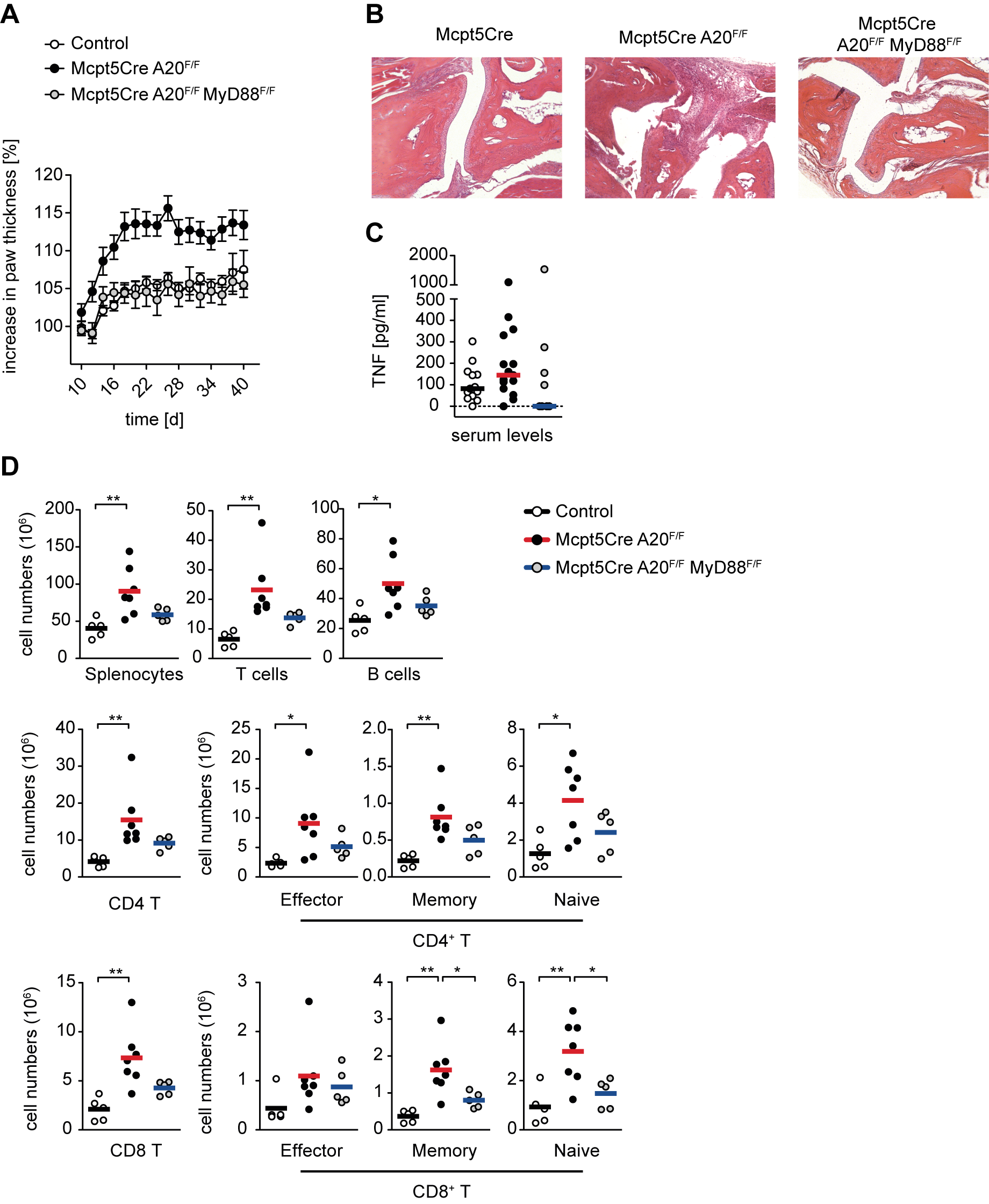

Supplement: Figure S4 — Mast cell-specific A20-deficient mice develop exaggerated CIA. (A) Increase in paw thickness during CIA was measured using an engineer's micrometer. Data are means ± SEM (for visual clarity instead of SD) from at least 10 mice per genotype (Control, 16 Mcpt5Cre mice). (B) Histological sections of ankle joints from CIA mice stained with hematoxylin and eosin. (C) Serum TNF levels in CIA mice were measured by ELISA. Bars indicate medians from at least 10 mice per genotype (Control, 13 Mcpt5Cre mice). (D) Scatter plots show absolute cell numbers of total splenocytes, B cells (B220+), T cells (TCRβ+), and CD4+ and CD8+ T cell (TCRβ+) subsets, and bars indicate means from at least five mice per genotype (Control, 5 Mcpt5Cre mice) (effector-like, CD44hiCD62Llo; memory-like, CD44hiCD62Lhi; naive, CD44lo-intCD62Lhi). *p<0.05, **p<0.01 (one-way ANOVA). (TIF) [file pbio.1001762.s004.tif]

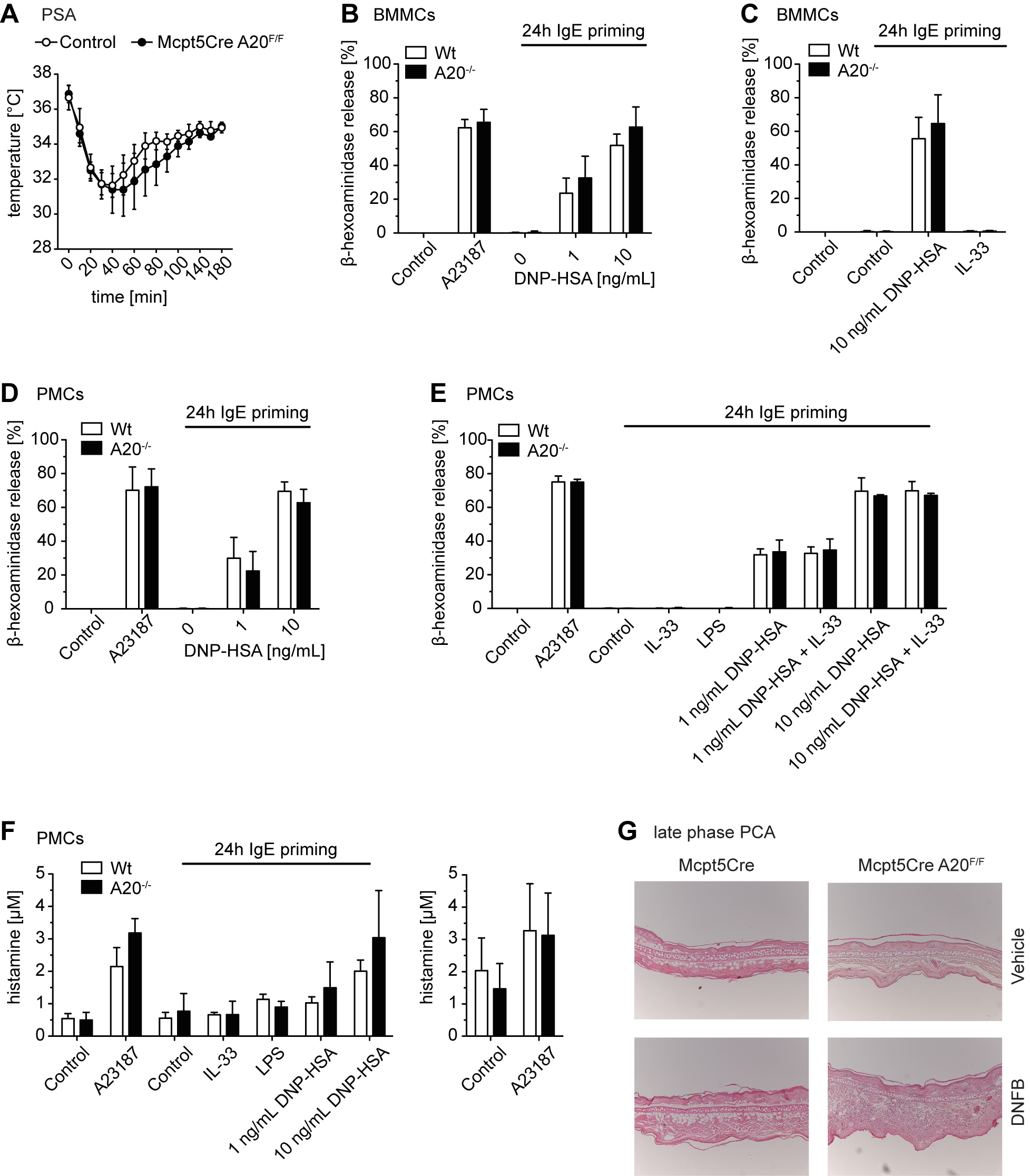

Supplement: Figure S5 — Normal degranulation and immediate but exacerbated late phase anaphylactic responses in the absence of A20. (A) Data show dorsal skin temperatures over time during PSA reactions measured with a thermography camera and are means ± SD from at least four mice per genotype (Control, 5 Cre− littermates). (B–E) BMMCs (B and C) and PMCs (D and E) were loaded for 24 h with 1 µg/mL anti-DNP IgE and subsequently stimulated for 30 min with the indicated concentrations of DNP–HSA, 10 ng/mL IL-33, 10 µg/mL LPS, or 500 ng/mL A23187. Degranulation was determined by measuring the activity of β-hexosaminidase in supernatants and cell lysates. (F) PMCs were loaded for 24 h with 1 µg/mL anti-DNP IgE and subsequently stimulated for 30 min with the indicated concentrations of DNP–HSA, 10 ng/mL IL-33, 10 µg/mL LPS, or 500 ng/mL A23187. Histamine release was measured by EIA. Two experiments are shown. Data are means + SD from six (B), five (C), five (D), three (E), or three (F) independent mast cell preparations. (G) Histological ear skin sections of late phase PCA reactions stained with hematoxylin and eosin. (TIF) [file pbio.1001762.s005.tif]

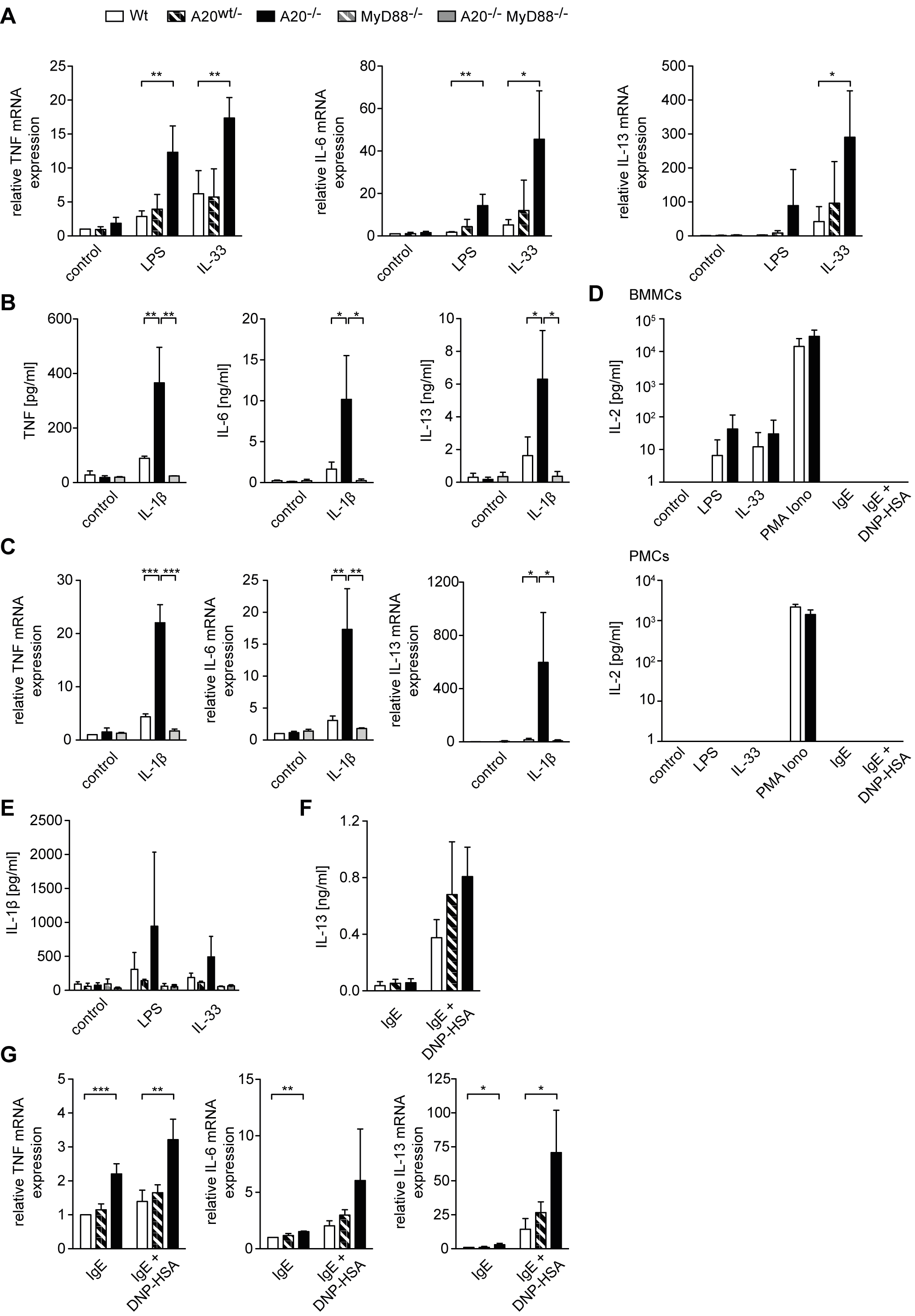

Supplement: Figure S6 — A20 is a key negative regulator of mast cell activation. (A) BMMCs were stimulated for 6 h with 10 µg/mL LPS or 10 ng/mL IL-33. TNF, IL-6, and IL-13 mRNA levels were determined by quantitative RT-PCR. Changes in transcript levels were calculated after normalization to PBGD. Data are means + SD from four independent experiments. *p<0.05, **p<0.01 (one-way ANOVA). (B) TNF, IL-6, and IL-13 secretion of BMMCs stimulated for 6 h with 10 ng/mL IL-1β were measured by ELISA. Data are means + SD from three independent experiments. *p<0.05, **p<0.01 (one-way repeated measures ANOVA). (C) BMMCs were stimulated as in (B). TNF, IL-6, and IL-13 mRNA levels were determined by quantitative RT-PCR. Changes in transcript levels were calculated after normalization to PBGD. Data are means + SD from three independent experiments. *p<0.05, **p<0.01, ***p<0.001 (one-way ANOVA). (D) BMMCs and PMCs were stimulated with 10 µg/mL LPS, 10 ng/mL IL-33, or 40 nM Phorbol-12-myristate-13-acetate (PMA) and 400 nM Ionomycin (Iono) for 6 h. BMMCs and PMCs were loaded for 2 h with 1 µg/mL anti-DNP IgE and subsequently stimulated for 6 h with 10 ng/mL DNP–HSA. IL-2 secretion was measured by ELISA. Data are means + SD from three independent experiments (BMMCs) or three independent mast cell preparations (PMCs). (E) IL-1β secretion of BMMCs stimulated for 6 h as in (A) were measured by ELISA. Data are means + SD from three independent experiments. (F) IL-13 secretion of BMMCs loaded for 2 h with 1 µg/mL anti-DNP IgE and subsequently stimulated for 6 h with 10 ng/mL DNP–HSA was measured by ELISA. Data are means + SD from three independent experiments. (G) BMMCs were stimulated as in (F). TNF, IL-6, and IL-13 mRNA levels were determined by quantitative RT-PCR. Changes in transcript levels were calculated after normalization to PBGD. Data are means + SD from three independent experiments. *p<0.05, **p<0.01, ***p<0.001 (one-way ANOVA). (TIF) [file pbio.1001762.s006.tif]
